# Linking essay-writing tests using many-facet models and neural automated essay scoring

**DOI:** 10.3758/s13428-024-02485-2

**Published:** 2024-08-20

**Authors:** Masaki Uto, Kota Aramaki

**Affiliations:** The University of Electro-Communications, Tokyo, Japan

**Keywords:** Writing assessment, Item response theory, Many-facet Rasch models, IRT linking, Automated essay scoring, Educational measurement

## Abstract

For essay-writing tests, challenges arise when scores assigned to essays are influenced by the characteristics of raters, such as rater severity and consistency. Item response theory (IRT) models incorporating rater parameters have been developed to tackle this issue, exemplified by the many-facet Rasch models. These IRT models enable the estimation of examinees’ abilities while accounting for the impact of rater characteristics, thereby enhancing the accuracy of ability measurement. However, difficulties can arise when different groups of examinees are evaluated by different sets of raters. In such cases, test linking is essential for unifying the scale of model parameters estimated for individual examinee–rater groups. Traditional test-linking methods typically require administrators to design groups in which either examinees or raters are partially shared. However, this is often impractical in real-world testing scenarios. To address this, we introduce a novel method for linking the parameters of IRT models with rater parameters that uses neural automated essay scoring technology. Our experimental results indicate that our method successfully accomplishes test linking with accuracy comparable to that of linear linking using few common examinees.

## Introduction

The growing demand for assessing higher-order skills, such as logical reasoning and expressive capabilities, has led to increased interest in essay-writing assessments (Abosalem, 2016; Bernardin et al., 2016; Liu et al., 2014; Rosen & Tager, 2014; Schendel & Tolmie, 2017). In these assessments, human raters assess the written responses of examinees to specific writing tasks. However, a major limitation of these assessments is the strong influence that rater characteristics, including severity and consistency, have on the accuracy of ability measurement (Bernardin et al., 2016; Eckes, 2005, 2023; Kassim, 2011; Myford & Wolfe, 2003). Several item response theory (IRT) models that incorporate parameters representing rater characteristics have been proposed to mitigate this issue (Eckes, 2023; Myford & Wolfe, 2003; Uto & Ueno, 2018).

The most prominent among them are many-facet Rasch models (MFRMs) (Linacre, 1989), and various extensions of MFRMs have been proposed to date (Patz & Junker, 1999; Patz et al., 2002; Uto & Ueno, 2018, 2020). These IRT models have the advantage of being able to estimate examinee ability while accounting for rater effects, making them more accurate than simple scoring methods based on point totals or averages.

However, difficulties can arise when essays from different groups of examinees are evaluated by different sets of raters, a scenario often encountered in real-world testing. For instance, in academic settings such as university admissions, individual departments may use different pools of raters to assess essays from specific applicant pools. Similarly, in the context of large-scale standardized tests, different sets of raters may be allocated to various test dates or locations. Thus, when applying IRT models with rater parameters to account for such real-world testing cases while also ensuring that ability estimates are comparable across groups of examinees and raters, *test linking* becomes essential for unifying the scale of model parameters estimated for each group.

Conventional test-linking methods generally require some overlap of examinees or raters across the groups being linked (Eckes, 2023; Engelhard, 1997; Ilhan, 2016; Linacre, 2014; Uto, 2021a). For example, linear linking based on common examinees, a popular linking method, estimates the IRT parameters for shared examinees using data from each group. These estimates are then used to build a linear regression model, which adjusts the parameter scales across groups. However, the design of such overlapping groups can often be impractical in real-world testing environments.

To facilitate test linking in these challenging environments, we introduce a novel method that leverages neural automated essay scoring (AES) technology. Specifically, we employ a cutting-edge deep neural AES method (Uto & Okano, 2021) that can predict IRT-based abilities from examinees’ essays. The central concept of our linking method is to construct an AES model using the ability estimates of examinees in a reference group, along with their essays, and then to apply this model to predict the abilities of examinees in other groups. An important point is that the AES model is trained to predict examinee abilities on the scale established by the reference group. This implies that the trained AES model can predict the abilities of examinees in other groups on the ability scale established by the reference group. Therefore, we use the predicted abilities to calculate the linking coefficients required for linear linking and to perform a test linking. In this study, we conducted experiments based on real-world data to demonstrate that our method successfully accomplishes test linking with accuracy comparable to that of linear linking using few common examinees.

It should be noted that previous studies have attempted to employ AES technologies for test linking (Almond, 2014; Olgar, 2015), but their focus has primarily been on linking tests with varied writing tasks or a mixture of essay tasks and objective items, while overlooking the influence of rater characteristics. This differs from the specific scenarios and goals that our study aims to address. To the best of our knowledge, this is the first study that employs AES technologies to link IRT models incorporating rater parameters for writing assessments without the need for common examinees and raters.

## Setting and data

In this study, we assume scenarios in which two groups of examinees respond to the same writing task and their written essays are assessed by two distinct sets of raters following the same scoring rubric. We refer to one group as the *reference group*, which serves as the basis for the scale, and the other as the *focal group*, whose scale we aim to align with that of the reference group.

Let $u^{\text {ref}}_{jr}$ be the score assigned by rater $r \in \mathcal {R}^{\text {ref}}$ to the essay of examinee $j \in \mathcal {J}^{\text {ref}}$, where $\mathcal {R}^{\text {ref}}$ and $\mathcal {J}^{\text {ref}}$ denote the sets of raters and examinees in the reference group, respectively. Then, a collection of scores for the reference group can be defined as

1 $$\begin{aligned} \textbf{U}^{\text {ref}} = \{ u^{\text {ref}}_{jr} \in \mathcal {K} \cup \{-1\} \mid j \in \mathcal {J}^{\text {ref}}, r \in \mathcal {R}^{\text {ref}} \}, \end{aligned}$$

where $\mathcal{K} = \{1,\ldots ,K\}$ represents the rating categories, and $-1$ indicates missing data.

Similarly, a collection of scores for the focal group can be defined as

2 $$\begin{aligned} \textbf{U}^{\text {foc}} = \{ u^{\text {foc}}_{jr} \in \mathcal {K} \cup \{-1\} \mid j \in \mathcal {J}^{\text {foc}}, r \in \mathcal {R}^{\text {foc}} \}, \end{aligned}$$

where $u^{\text {foc}}_{jr}$ indicates the score assigned by rater $r \in \mathcal {R}^{\text {foc}}$ to the essay of examinee $j \in \mathcal {J}^{\text {foc}}$, and $\mathcal {R}^{\text {foc}}$ and $\mathcal {J}^{\text {foc}}$ represent the sets of raters and examinees in the focal group, respectively.

The primary objective of this study is to apply IRT models with rater parameters to the two sets of data, $\textbf{U}^{\text {ref}}$ and $\textbf{U}^{\text {foc}}$, and to establish IRT parameter linking without shared examinees and raters: $\mathcal {J}^{\text {ref}} \cap \mathcal {J}^{\text {foc}} = \emptyset$ and $\mathcal {R}^{\text {ref}} \cap \mathcal {R}^{\text {foc}} = \emptyset$. More specifically, we seek to align the scale derived from $\textbf{U}^{\text {foc}}$ with that of $\textbf{U}^{\text {ref}}$.

## Item response theory

IRT (Lord, 1980), a test theory grounded in mathematical models, has recently gained widespread use in various testing situations due to the growing prevalence of computer-based testing. In objective testing contexts, IRT makes use of latent variable models, commonly referred to as IRT models. Traditional IRT models, such as the Rasch model and the two-parameter logistic model, give the probability of an examinee’s response to a test item as a probabilistic function influenced by both the examinee’s latent ability and the item’s characteristic parameters, such as difficulty and discrimination. These IRT parameters can be estimated from a dataset consisting of examinees’ responses to test items.

However, traditional IRT models are not directly applicable to essay-writing test data, where the examinees’ responses to test items are assessed by multiple human raters. Extended IRT models with rater parameters have been proposed to address this issue (Eckes, 2023; Jin and Wang, 2018; Linacre, 1989; Shin et al., 2019; Uto, 2023; Wilson & Hoskens, 2001).

### Many-facet Rasch models and their extensions

The MFRM (Linacre, 1989) is the most commonly used IRT model that incorporates rater parameters. Although several variants of the MFRM exist (Eckes, 2023; Myford & Wolfe, 2004), the most representative model defines the probability that the essay of examinee *j* for a given test item (either a writing task or prompt) *i* receives a score of *k* from rater *r* as

3 $$\begin{aligned} P_{ijrk} = \frac{\exp \sum _{m = 1}^{k}\left[ D(\theta _j-\beta _i-\beta _{r} - d_{m}) \right] }{\sum _{l = 1}^{K} \exp \sum _{m = 1}^{l}\left[ D(\theta _j-\beta _i-\beta _{r} - d_{m}) \right] }, \end{aligned}$$

where $\theta _j$ is the latent ability of examinee *j*, $\beta _{i}$ represents the difficulty of item *i*, $\beta _{r}$ represents the severity of rater *r*, and $d_{m}$ is a step parameter denoting the difficulty of transitioning between scores $m-1$ and *m*. $D = 1.7$ is a scaling constant used to minimize the difference between the normal and logistic distribution functions. For model identification, $\sum _{i} \beta _{i} = 0$, $d_1 = 0$, $\sum _{m = 2}^{K} d_{m} = 0$, and a normal distribution for the ability $\theta _j$ are assumed.

Another popular MFRM is one in which $d_{m}$ is replaced with $d_{rm}$, a rater-specific step parameter denoting the severity of rater *r* when transitioning from score $m-1$ to *m*. This model is often used to investigate variations in rating scale criteria among raters caused by differences in the central tendency, extreme response tendency, and range restriction among raters (Eckes, 2023; Myford & Wolfe, 2004; Qiu et al., 2022; Uto, 2021a).

A recent extension of the MFRM is the generalized many-facet model (GMFM) (Uto & Ueno, 2020)^1^, which incorporates parameters denoting rater consistency and item discrimination. GMFM defines the probability $P_{ijrk}$ as

4 $$\begin{aligned} P_{ijrk} = \frac{\exp \sum _{m = 1}^{k}\left[ D\alpha _i\alpha _r(\theta _j-\beta _i-\beta _{r} - d_{rm}) \right] }{\sum _{l = 1}^{K} \exp \sum _{m = 1}^{l}\left[ D\alpha _i\alpha _r(\theta _j-\beta _i-\beta _{r} - d_{rm}) \right] }, \end{aligned}$$

where $\alpha _i$ indicates the discrimination power of item *i*, and $\alpha _r$ indicates the consistency of rater *r*. For model identification, $\prod _{r} \alpha _i = 1$, $\sum _{i} \beta _{i} = 0$, $d_{r1} = 0$, $\sum _{m = 2}^{K} d_{rm} = 0$, and a normal distribution for the ability $\theta _j$ are assumed.

In this study, we seek to apply the aforementioned IRT models to data involving a single test item, as detailed in the *Setting and data* section. When there is only one test item, the item parameters in the above equations become superfluous and can be omitted. Consequently, the equations for these models can be simplified as follows.

- MFRM:
  5 $$\begin{aligned} P_{ijrk} = \frac{\exp \sum _{m = 1}^{k}\left[ D(\theta _j-\beta _{r} - d_{m}) \right] }{\sum _{l = 1}^{K} \exp \sum _{m = 1}^{l}\left[ D(\theta _j-\beta _{r} - d_{m}) \right] }, \end{aligned}$$
- MFRM with rater-specific step parameters (referred to as *MFRM with RSS* in the subsequent sections):
  6 $$\begin{aligned} P_{ijrk} = \frac{\exp \sum _{m = 1}^{k}\left[ D(\theta _j-\beta _{r} - d_{rm}) \right] }{\sum _{l = 1}^{K} \exp \sum _{m = 1}^{l}\left[ D(\theta _j-\beta _{r} - d_{rm}) \right] }, \end{aligned}$$
- GMFM:
  7 $$\begin{aligned} P_{ijrk} = \frac{\exp \sum _{m = 1}^{k}\left[ D\alpha _r(\theta _j-\beta _{r} - d_{rm}) \right] }{\sum _{l = 1}^{K} \exp \sum _{m = 1}^{l}\left[ D\alpha _r(\theta _j-\beta _{r} - d_{rm}) \right] }. \end{aligned}$$

Note that the GMFM can simultaneously capture the following typical characteristics of raters, whereas the MFRM and MFRM with RSS can only consider a subset of these characteristics.

- *Severity*: This refers to the tendency of some raters to systematically assign higher or lower scores compared with other raters regardless of the actual performance of the examinee. This tendency is quantified by the parameter $\beta _r$.
- *Consistency*: This is the extent to which raters maintain their scoring criteria consistently over time and across different examinees. Consistent raters exhibit stable scoring patterns, which make their evaluations more reliable and predictable. In contrast, inconsistent raters show varying scoring tendencies. This characteristic is represented by the parameter $\alpha _r$.
- *Range Restriction*: This describes the limited variability in scores assigned by a rater. Central tendency and extreme response tendency are special cases of range restriction. This characteristic is represented by the parameter $d_{rm}$.

For details on how these characteristics are represented in the GMFM, see the article (Uto & Ueno, 2020).

Based on the above, it is evident that both the MFRM and MFRM with RSS are special cases of the GMFM. Specifically, the GMFM with constant rater consistency corresponds to the MFRM with RSS. Moreover, the MFRM with RSS that assumes no differences in the range restriction characteristic among raters aligns with the MFRM.

### Linking

When the aforementioned IRT models are applied to datasets from multiple groups composed of different examinees and raters, such as $\textbf{U}^{\text {red}}$ and $\textbf{U}^{\text {foc}}$, the scales of the estimated parameters generally differ among them. This discrepancy arises because IRT permits arbitrary scaling of parameters for each independent dataset. An exception occurs when it is feasible to assume equality in between-test distributions of examinee abilities and rater parameters (Linacre, 2014). However, real-world testing conditions may not always satisfy this assumption. Therefore, if the aim is to compare parameter estimates between different groups, test linking is generally required to unify the scale of model parameters estimated from each individual group’s dataset.

One widely used approach for test linking is *linear linking*. In the context of the essay-writing test considered in this study, implementing linear linking necessitates designing two groups so that there is some overlap in examinees between them. With this design, IRT parameters for the shared examinees are estimated individually for each group. These estimates are then used to construct a linear regression model for aligning the parameter scales across groups, thereby rendering them comparable. We now introduce the *mean and sigma method* (Kolen & Brennan, 2014; Marco, 1977), a popular method for linear linking, and illustrate the procedures for parameter linking specifically for the GMFM, as defined in Eq. 7, because both the MFRM and the MFRM with RSS can be regarded as special cases of the GMFM, as explained earlier.

To elucidate this, let us assume that the datasets corresponding to the reference and focal groups, denoted as $\textbf{U}^{\text {ref}}$ and $\textbf{U}^{\text {foc}}$, contain overlapping sets of examinees. Furthermore, let us assume that $\hat{\varvec{\theta }}^{\text {foc}}$, $\hat{\varvec{\alpha }}^{\text {foc}}$, $\hat{\varvec{\beta }}^{\text {foc}}$, and $\hat{\varvec{d}}^{\text {foc}}$ are the GMFM parameters estimated from $\textbf{U}^{\text {foc}}$. The mean and sigma method aims to transform these parameters linearly so that their scale aligns with those estimated from $\textbf{U}^{\text {ref}}$. This transformation is guided by the equations

8 $$\begin{aligned} \begin{aligned} \tilde{\varvec{\theta }}^{\text {foc}}&= A\hat{\varvec{\theta }}^{\text {foc}} + K, \\ \tilde{\varvec{\alpha }}^{\text {foc}}&= \frac{\hat{\varvec{\alpha }}^{\text {foc}}}{A}, \\ \tilde{\varvec{\beta }}^{\text {foc}}&= A\hat{\varvec{\beta }}^{\text {foc}} + K, \\ \tilde{\varvec{d}}^{\text {foc}}&= A\hat{\varvec{d}}^{\text {foc}}, \end{aligned} \end{aligned}$$

where $\tilde{\varvec{\theta }}^{\text {foc}}$, $\tilde{\varvec{\alpha }}^{\text {foc}}$, $\tilde{\varvec{\beta }}^{\text {foc}}$, and $\tilde{\varvec{d}}^{\text {foc}}$ represent the scale-transformed parameters for the focal group. The linking coefficients are defined as

9 $$\begin{aligned} \begin{aligned} A&= \frac{{\sigma }^{\text {ref}}}{{\sigma }^{\text {foc}}},\\ K&= {\mu }^{\text {ref}} - A{\mu }^{\text {foc}}, \end{aligned} \end{aligned}$$

where ${\mu }^{\text {ref}}$ and ${\sigma }^{\text {ref}}$ represent the mean and standard deviation (SD) of the common examinees’ ability values estimated from $\textbf{U}^{\text {ref}}$, and ${\mu }^{\text {foc}}$ and ${\sigma }^{\text {foc}}$ represent those values obtained from $\textbf{U}^{\text {foc}}$.

This linear linking method is applicable when there are common examinees across different groups. However, as discussed in the introduction, arranging for multiple groups with partially overlapping examinees (and/or raters) can often be impractical in real-world testing environments. To address this limitation, we aim to facilitate test linking without the need for common examinees and raters by leveraging AES technology.

## Automated essay scoring models

Many AES methods have been developed over recent decades and can be broadly categorized into either *feature-engineering* or *automatic feature extraction* approaches (Hussein et al., 2019; Ke & Ng, 2019). The feature-engineering approach predicts essay scores using either a regression or classification model that employs manually designed features, such as essay length and the number of spelling errors (Amorim et al., 2018; Dascalu et al., 2017; Nguyen & Litman, 2018; Shermis & Burstein, 2002). The advantages of this approach include greater interpretability and explainability. However, it generally requires considerable effort in developing effective features to achieve high scoring accuracy for various datasets. Automatic feature extraction approaches based on deep neural networks (DNNs) have recently attracted attention as a means of eliminating the need for feature engineering. Many DNN-based AES models have been proposed in the last decade and have achieved state-of-the-art accuracy (Alikaniotis et al., 2016; Dasgupta et al., 2018; Farag et al., 2018; Jin et al., 2018; Mesgar & Strube, 2018; Mim et al., 2019; Nadeem et al., 2019; Ridley et al., 2021; Taghipour & Ng, 2016; Uto, 2021b; Wang et al., 2018). In the next section, we introduce the most widely used DNN-based AES model, which utilizes Bidirectional Encoder Representations from Transformers (BERT) (Devlin et al., 2019).

### BERT-based AES model

BERT, a pre-trained language model developed by Google’s AI language team, achieved state-of-the-art performance in various natural language processing (NLP) tasks in 2019 (Devlin et al., 2019). Since then, it has frequently been applied to AES (Rodriguez et al., 2019) and automated short-answer grading (Liu et al., 2019; Lun et al., 2020; Sung et al., 2019) and has demonstrated high accuracy.

BERT is structured as a multilayer bidirectional transformer network, where the transformer is a neural network architecture designed to handle ordered sequences of data using an attention mechanism. See Ref. (Vaswani et al., 2017) for details of transformers.

BERT undergoes training in two distinct phases, *pretraining* and *fine-tuning*. The pretraining phase utilizes massive volumes of unlabeled text data and is conducted through two unsupervised learning tasks, specifically, *masked language modeling* and *next-sentence prediction*. Masked language modeling predicts the identities of words that have been masked out of the input text, while next-sequence prediction predicts whether two given sentences are adjacent.

**Fig. 1:**
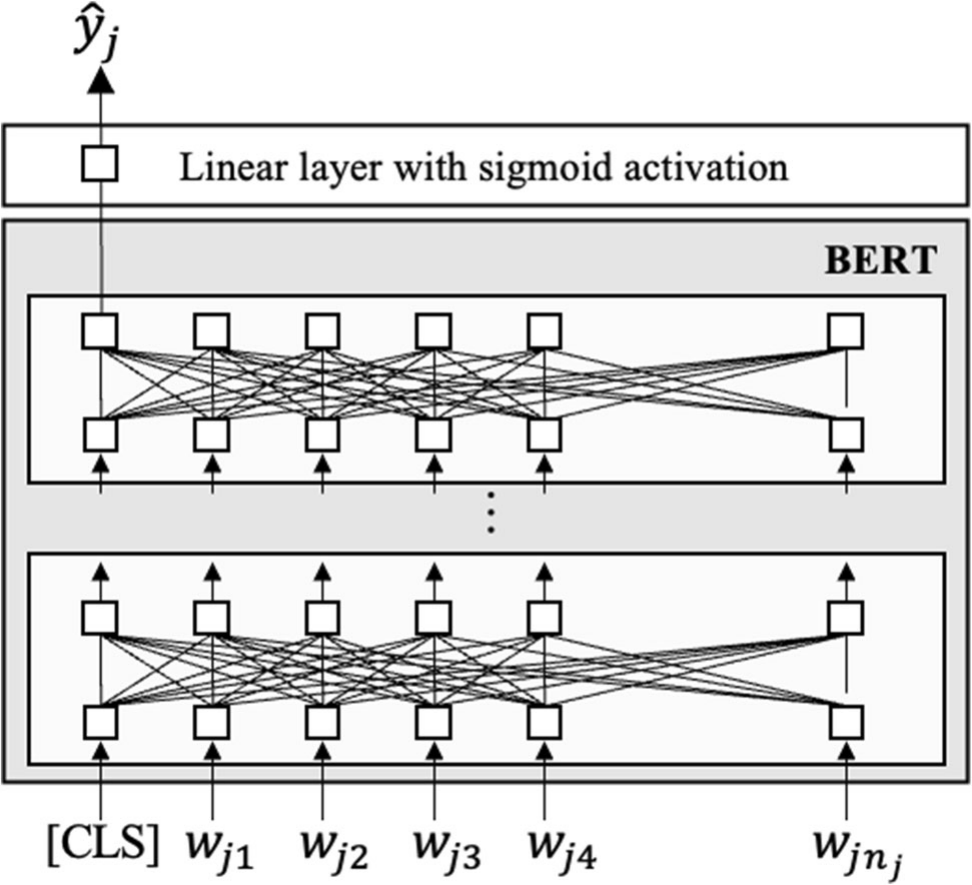
BERT-based AES model architecture. $w_{jt}$ is the *t*-th word in the essay of examinee *j*, $n_j$ is the number of words in the essay, and $\hat{y}_{j}$ represents the predicted score from the model

Fine-tuning is required to adapt a pre-trained BERT model for a specific NLP task, including AES. This entails retraining the BERT model using a task-specific supervised dataset after initializing the model parameters with pre-trained values and augmenting with task-specific output layers. For AES applications, the addition of a special token, **[CLS]**, at the beginning of each input is required. Then, BERT condenses the entire input text into a fixed-length real-valued hidden vector referred to as the *distributed text representation*, which corresponds to the output of the special token **[CLS]** (Devlin et al., 2019). AES scores can thus be derived by feeding the distributed text representation into a *linear layer with sigmoid activation*, as depicted in Fig. 1. More formally, let $\varvec{h}$ be the distributed text representation. The linear layer with sigmoid activation is defined as $\sigma (\varvec{W}\varvec{h}+\text{ b})$, where $\varvec{W}$ is a weight matrix and $\text{ b }$ is a bias, both learned during the fine-tuning process. The sigmoid function $\sigma ()$ maps its input to a value between 0 and 1. Therefore, the model is trained to minimize an error loss function between the predicted scores and the gold-standard scores, which are normalized to the [0, 1] range. Moreover, score prediction using the trained model is performed by linearly rescaling the predicted scores back to the original score range.

### Problems with AES model training

As mentioned above, to employ BERT-based and other DNN-based AES models, they must be trained or fine-tuned using a large dataset of essays that have been graded by human raters. Typically, the mean-squared error (MSE) between the predicted and the gold-standard scores serves as the loss function for model training. Specifically, let $y_{j}$ be the normalized gold-standard score for the *j*-th examinee’s essay, and let $\hat{y}_{j}$ be the predicted score from the model. The MSE loss function is then defined as

10 $$\begin{aligned} \frac{1}{J}\sum _{j=1}^J(y_{j}-\hat{y}_{j})^2, \end{aligned}$$

where *J* denotes the number of examinees, which is equivalent to the number of essays, in the training dataset.

Here, note that a large-scale training dataset is often created by assigning a few raters from a pool of potential raters to each essay to reduce the scoring burden and to increase scoring reliability. In such cases, the gold-standard score for each essay is commonly determined by averaging the scores given by multiple raters assigned to that essay. However, as discussed in earlier sections, these straightforward average scores are highly sensitive to rater characteristics. When training data includes rater bias effects, an AES model trained on that data can show decreased performance as a result of inheriting these biases (Amorim et al., 2018; Huang et al., 2019; Li et al., 2020; Wind et al., 2018). An AES method that uses IRT has been proposed to address this issue (Uto & Okano, 2021).

### AES method using IRT

**Fig. 2:**
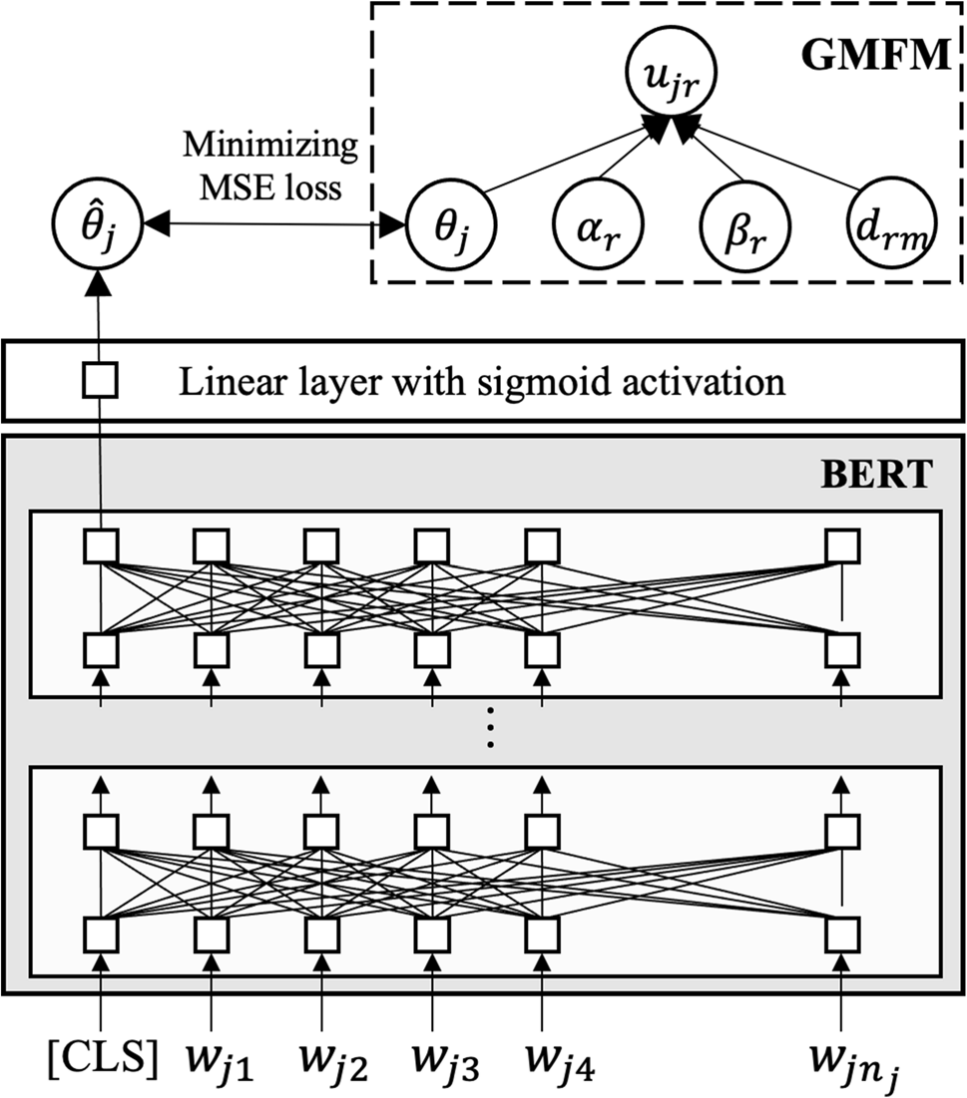
Architecture of a BERT-based AES model that uses IRT

The main idea behind the AES method using IRT (Uto & Okano, 2021) is to train an AES model using the ability value $\theta _j$ estimated by IRT models with rater parameters, such as MFRM and its extensions, from the data given by multiple raters for each essay, instead of a simple average score. Specifically, AES model training in this method occurs in two steps, as outlined in Fig. 2.

1. Estimate the IRT-based abilities $\varvec{\theta }$ from a score dataset, which includes scores given to essays by multiple raters.
2. Train an AES model given the ability estimates as the gold-standard scores. Specifically, the MSE loss function for training is defined as
  11 $$\begin{aligned} \frac{1}{J}\sum _{j=1}^J(\theta _{j}-\hat{\theta }_{j})^2, \end{aligned}$$
  where $\hat{\theta }_j$ represents the AES’s predicted ability of the *j*-th examinee, and $\theta _{j}$ is the gold-standard ability for the examinee obtained from Step 1. Note that the gold-standard scores are rescaled into the range [0, 1] by applying a linear transformation from the logit range $[-3, 3]$ to [0, 1]. See the original paper (Uto & Okano, 2021) for details.

A trained AES model based on this method will not reflect bias effects because IRT-based abilities $\varvec{\theta }$ are estimated while removing rater bias effects.

In the prediction phase, the score for an essay from examinee $j^{\prime }$ is calculated in two steps.

1. Predict the IRT-based ability $\theta _{j^{\prime }}$ for the examinee using the trained AES model, and then linearly rescale it to the logit range $[-3, 3]$.
2. Calculate the expected score $\mathbb {E}_{r,k}\left[ P_{j^{\prime }rk}\right]$, which corresponds to an unbiased original-scaled score, given $\theta _{j'}$ and the rater parameters. This is used as a predicted essay score in this method.

This method originally aimed to train an AES model while mitigating the impact of varying rater characteristics present in the training data. A key feature, however, is its ability to predict an examinee’s IRT-based ability from their essay texts. Our linking approach leverages this feature to enable test linking without requiring common examinees and raters.

**Fig. 3:**
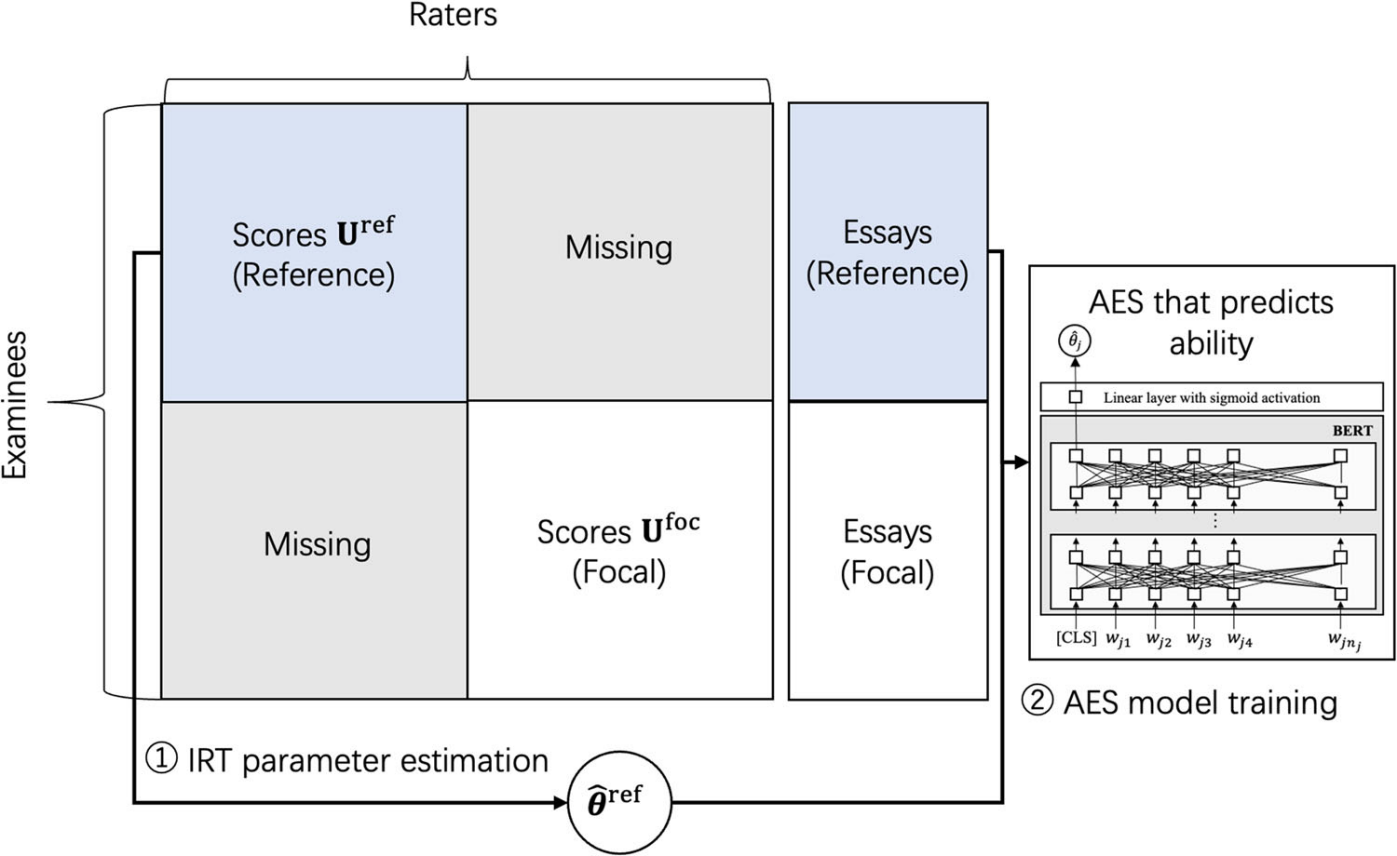
Outline of our proposed method, steps 1 and 2

**Fig. 4:**
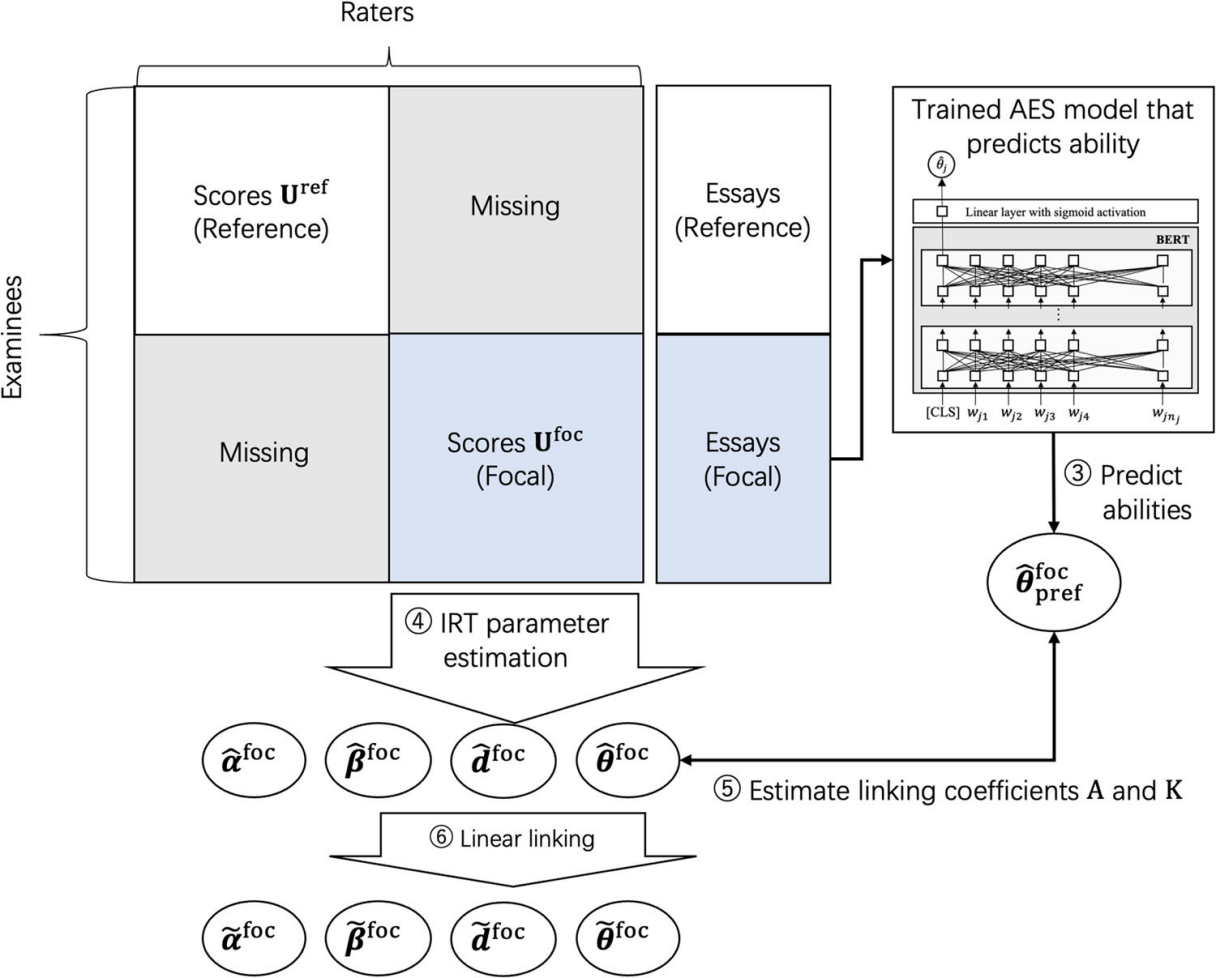
Outline of our proposed method, steps 3–6

## Proposed method

The core idea behind our method is to develop an AES model that predicts examinee ability using score and essay data from the reference group, and then to use this model to predict the abilities of examinees in the focal group. These predictions are then used to estimate the linking coefficients for a linear linking. An outline of our method is illustrated in Figs. 3 and 4. The detailed steps involved in the procedure are as follows.

1. Estimate the IRT model parameters from the reference group’s data $\textbf{U}^{\text {ref}}$ to obtain $\hat{\varvec{\theta }}^{\text {ref}}$ indicating the ability estimates of the examinees in the reference group.
2. Use the ability estimates $\hat{\varvec{\theta }}^{\text {ref}}$ and the essays written by the examinees in the reference group to train the AES model that predicts examinee ability.
3. Use the trained AES model to predict the abilities of examinees in the focal group by inputting their essays. We designate these AES-predicted abilities as $\hat{\varvec{\theta }}^{\text {foc}}_{\text {pred}}$ from here on. An important point to note is that the AES model is trained to predict ability values on the parameter scale aligned with the reference group’s data, meaning that the predicted abilities for examinees in the focal group follow the same scale.
4. Estimate the IRT model parameters from the focal group’s data $\textbf{U}^{\text {foc}}$.
5. Calculate the linking coefficients *A* and *K* using the AES-predicted abilities $\hat{\varvec{\theta }}^{\text {foc}}_{\text {pred}}$ and the IRT-based ability estimates $\hat{\varvec{\theta }}^{\text {foc}}$ for examinees in the focal group as follows.
  12 $$\begin{aligned} \begin{aligned} A&= \frac{{\sigma }^{\text {foc}}_{\text {pred}}}{{\sigma }^{\text {foc}}}, \\ K&= {\mu }^{\text {foc}}_{\text {pred}} - A{\mu }^{\text {foc}}, \end{aligned} \end{aligned}$$
  where ${\mu }^{\text {foc}}_{\text {pred}}$ and ${\sigma }^{\text {foc}}_{\text {pred}}$ represent the mean and the SD of the AES-predicted abilities $\hat{\varvec{\theta }}^{\text {foc}}_{\text {pred}}$, respectively. Furthermore, ${\mu }^{\text {foc}}$ and ${\sigma }^{\text {foc}}$ represent the corresponding values for the IRT-based ability estimates $\hat{\varvec{\theta }}^{\text {foc}}$.
6. Apply linear linking based on the mean and sigma method given in Eq. 8 using the above linking coefficients and the parameter estimates for the focal group obtained in Step 4. This procedure yields parameter estimates for the focal group that are aligned with the scale of the parameters of the reference group.

As described in Step 3, the AES model used in our method is trained to predict examinee abilities on the scale derived from the reference data $\textbf{U}^{\text {ref}}$. Therefore, the abilities predicted by the trained AES model for the examinees in the focal group, denoted as $\hat{\varvec{\theta }}^{\text {foc}}_{\text {pred}}$, also follow the ability scale derived from the reference data. Consequently, by using the AES-predicted abilities, we can infer the differences in the ability distribution between the reference and focal groups. This enables us to estimate the linking coefficients, which then allows us to perform linear linking based on the mean and sigma method. Thus, our method allows for test linking without the need for common examinees and raters.

It is important to note that the current AES model for predicting examinees’ abilities does not necessarily offer sufficient prediction accuracy for individual ability estimates. This implies that their direct use in mid- to high-stakes assessments could be problematic. Therefore, we focus solely on the mean and SD values of the ability distribution based on predicted abilities, rather than using individual predicted ability values. Our underlying assumption is that these AES models can provide valuable insights into differences in the ability distribution across various groups, even though the individual predictions might be somewhat inaccurate, thereby substantiating their utility for test linking.

## Experiments

In this section, we provide an overview of the experiments we conducted using actual data to evaluate the effectiveness of our method.

### Actual data

We used the dataset previously collected in Uto and Okano (2021). It consists of essays written in English by 1805 students from grades 7 to 10 along with scores from 38 raters for these essays. The essays originally came from the ASAP (Automated Student Assessment Prize) dataset, which is a well-known benchmark dataset for AES studies. The raters were native English speakers recruited from Amazon Mechanical Turk (AMT), a popular crowdsourcing platform. To alleviate the scoring burden, only a few raters were assigned to each essay, rather than having all raters evaluate every essay. Rater assignment was conducted based on a *systematic links design* (Shin et al., 2019; Uto, 2021a; Wind & Jones, 2019) to achieve IRT-scale linking. Consequently, each rater evaluated approximately 195 essays, and each essay was graded by four raters on average. The raters were asked to grade the essays using a holistic rubric with five rating categories, which is identical to the one used in the original ASAP dataset. The raters were provided no training before the scoring process began. The average Pearson correlation between the scores from AMT raters and the ground-truth scores included in the original ASAP dataset was 0.70 with an SD of 0.09. The minimum and maximum correlations were 0.37 and 0.81, respectively. Furthermore, we also calculated the intraclass correlation coefficient (ICC) between the scores from each AMT rater and the ground-truth scores. The average ICC was 0.60 with an SD of 0.15, and the minimum and maximum ICCs were 0.29 and 0.79, respectively. The calculation of the correlation coefficients and ICC for each AMT rater excluded essays that the AMT rater did not assess. Furthermore, because the ground-truth scores were given as the total scores from two raters, we divided them by two in order to align the score scale with the AMT raters’ scores.

For further analysis, we also evaluated the ICC among the AMT raters as their interrater reliability. In this analysis, missing value imputation was required because all essays were evaluated by a subset of AMT raters. Thus, we first applied multiple imputation with predictive mean matching to the AMT raters’ score dataset. In this process, we generated five imputed datasets. For each imputed dataset, we calculated the ICC among all AMT raters. Finally, we aggregated the ICC values from each imputed dataset to calculate the mean ICC and its SD. The results revealed a mean ICC of 0.43 with an SD of 0.01.

These results suggest that the reliability of raters is not necessarily high. This variability in scoring behavior among raters underscores the importance of applying IRT models with rater parameters. For further details of the dataset see Uto and Okano (2021).

### Experimental procedures

Using this dataset, we conducted the following experiment for three IRT models with rater parameters, MFRM, MFRM with RSS, and GMFM, defined by Eqs. 5, 6, and 7, respectively.

1. We estimated the IRT parameters from the dataset using the No-U-Turn sampler-based Markov chain Monte Carlo (MCMC) algorithm, given the prior distributions $\theta _j, \beta _r, d_m, d_{rm} \sim N(0, 1)$, and $\alpha _r \sim LN(0, 0.5)$ following the previous work (Uto & Ueno, 2020). Here, $N(\cdot , \cdot )$ and $LN(\cdot , \cdot )$ indicate normal and log-normal distributions with mean and SD values, respectively. The expected a posteriori (EAP) estimator was used as the point estimates.
2. We then separated the dataset randomly into two groups, the reference group and the focal group, ensuring no overlap of examinees and raters between them. In this separation, we selected examinees and raters in each group to ensure distinct distributions of examinee abilities and rater severities. Various separation patterns were tested and are listed in Table 1. For example, condition 1 in Table 1 means that the reference group comprised randomly selected high-ability examinees and low-severity raters, while the focal group comprised low-ability examinees and high-severity raters. Condition 2 provided a similar separation but controlled for narrower variance in rater severity in the focal group. Details of the group creation procedures can be found in Appendix A.
3. Using the obtained data for the reference and focal groups, we conducted test linking using our method, the details of which are given in the *Proposed method* section. In it, the IRT parameter estimations were carried out using the same MCMC algorithm as in Step 1.
4. We calculated the Root Mean Squared Error (RMSE) between the IRT parameters for the focal group, which were linked using our proposed method, and their gold-standard parameters. In this context, the gold-standard parameters were obtained by transforming the scale of the parameters estimated from the entire dataset in Step 1 so that it aligned with that of the reference group. Specifically, we estimated the IRT parameters using data from the reference group and collected those estimated from the entire dataset in Step 1. Then, using the examinees in the reference group as common examinees, we applied linear linking based on the mean and sigma method to adjust the scale of the parameters estimated from the entire dataset to match that of the reference group.
5. For comparison, we also calculated the RMSE between the focal group’s IRT parameters, obtained without applying the proposed linking, and their gold-standard parameters. This functions as the worst baseline against which the results of the proposed method are compared. Additionally, we examined other baselines that use linear linking based on common examinees. For these baselines, we randomly selected five or ten examinees from the reference group, who were assigned scores by at least two focal group’s raters in the entire dataset. The scores given to these selected examinees by the focal group’s raters were then merged with the focal group’s data, where the added examinees worked as common examinees between the reference and focal groups. Using this data, we examined linear linking using common examinees. Specifically, we estimated the IRT parameters from the data of the focal group with common examinees and applied linear linking based on the mean and sigma method using the ability estimates of the common examinees to align its scale with that of the reference group. Finally, we calculated the RMSE between the linked parameter estimates for the examinees and raters belonging only to the original focal group and their gold-standard parameters. Note that this common examinee approach operates under more advantageous conditions compared with the proposed linking method because it can utilize larger samples for estimating the parameters of raters in the focal group.
6. We repeated Steps 2–5 ten times for each data separation condition and calculated the average RMSE for four cases: one in which our proposed linking method was applied, one without linking, and two others where linear linkings using five and ten common examinees were applied.

**Table 1:** Data separation conditions

	Groups	Ability $\theta _j$		Severity $\beta _r$	
		Mean	Variance	Mean	Variance
Condition 1	Reference	High	Wide	Low	Wide
	Focal	Low	Wide	High	Wide
Condition 2	Reference	High	Wide	Low	Wide
	Focal	Low	Narrow	High	Narrow
Condition 3	Reference	High	Wide	High	Wide
	Focal	Low	Wide	Low	Wide
Condition 4	Reference	High	Wide	High	Wide
	Focal	Low	Narrow	Low	Wide
Condition 5	Reference	Moderate	Wide	Moderate	Wide
	Focal	Very high	Narrow	Very high	Narrow
Condition 6	Reference	Moderate	Wide	Moderate	Wide
	Focal	High	Narrow	High	Narrow
Condition 7	Reference	Moderate	Wide	Moderate	Wide
	Focal	Low	Narrow	Low	Narrow
Condition 8	Reference	Moderate	Wide	Moderate	Wide
	Focal	High	Narrow	Low	Narrow

The parameter estimation program utilized in Steps 1, 4, and 5 was implemented using RStan (Stan Development Team, 2018). The EAP estimates were calculated as the mean of the parameter samples obtained from 2,000 to 5,000 periods using three independent chains. The AES model was developed in Python, leveraging the PyTorch library^2^. For the AES model training in Step 3, we randomly selected $90\%$ of the data from the reference group to serve as the training set, with the remaining $10\%$ designated as the development set. We limited the maximum number of steps for training the AES model to 800 and set the maximum number of epochs to 800 divided by the number of mini-batches. Additionally, we employed early stopping based on the performance on the development set. The AdamW optimization algorithm was used, and the mini-batch size was set to 8.

### MCMC statistics and model fitting

Before delving into the results of the aforementioned experiments, we provide some statistics related to the MCMC-based parameter estimation. Specifically, we computed the Gelman–Rubin statistic $\hat{R}$ (Gelman et al., 2013; Gelman & Rubin, 1992), a well-established diagnostic index for convergence, as well as the effective sample size (ESS) and the number of divergent transitions for each IRT model during the parameter estimation phase in Step 1. Across all models, the $\hat{R}$ statistics were below 1.1 for all parameters, indicating convergence of the MCMC runs. Furthermore, as shown in the first row of Table 2, our ESS values for all parameters in all models exceeded the criterion of 400, which is considered sufficiently large according to Zitzmann and Hecht (2019). We also observed no divergent transitions in any of the cases. These results support the validity of the MCMC-based parameter estimation.

**Table 2:** Model selection criteria and MCMC statistics

	MFRM	MFRM with RSS	GMFM
ESS (Min)	3103.84	4299.31	1180.42
*PPP*	1.000	1.000	0.563
WAIC	14,864.17	14,404.58	**13,743.09**
WBIC	11,320.84	11,451.80	**11,028.14**

*Note:* ESS, effective sample size; PPP, posterior predictive *p* value

Furthermore, we evaluated the model – data fit for each IRT model during the parameter estimation step in Step 1. To assess this fit, we employed the posterior predictive *p* value (*PPP*-value) (Gelman et al., 2013), a commonly used metric for evaluating the model–data fit in Bayesian frameworks (Nering & Ostini, 2010; van der Linden, 2016). Specifically, we calculated the *PPP*-value using an averaged standardized residual, a conventional metric for IRT model fit in non-Bayesian settings, as a discrepancy function, similar to the approach in Nering and Ostini (2010); Tran (2020); Uto and Okano (2021). A well-fitted model yields a *PPP*-value close to 0.5, while poorly fitted models exhibit extreme low or high values, such as those below 0.05 or above 0.95. Additionally, we calculated two information criteria, the widely applicable information criterion (WAIC) (Watanabe, 2010) and the widely applicable Bayesian information criterion (WBIC) (Watanabe, 2013). The model that minimizes these criteria is considered optimal.

**Table 3:** RMSEs of the different experimental conditions for GMFM

		Conditions							
		1	2	3	4	5	6	7	8
Unlinked	$\theta _j$	0.77	0.79	0.80	0.80	0.65	0.44	0.45	0.46
	$\beta _r$	0.75	0.76	0.78	0.71	0.67	0.52	0.26	0.54
	$d_{r2}$	0.17	0.45	0.16	0.39	0.36	0.34	0.41	0.42
	$d_{r3}$	0.19	0.28	0.18	0.30	0.28	0.38	0.29	0.37
	$d_{r4}$	0.15	0.22	0.14	0.20	0.21	0.21	0.21	0.29
	$d_{r5}$	0.22	0.53	0.19	0.49	0.21	0.38	0.41	0.32
	$\alpha _r$	0.48	0.72	0.48	0.62	0.54	0.84	0.70	0.71
Linked by	$\theta _j$	0.45	0.35	0.45	0.31	0.30	0.27	0.29	0.28
proposed method	$\beta _r$	0.42	0.30	0.43	0.25	0.14	0.16	0.16	0.15
	$d_{r2}$	0.22	0.23	0.25	0.23	0.47	0.37	0.29	0.44
	$d_{r3}$	0.18	0.23	0.20	0.20	0.23	0.29	0.24	0.30
	$d_{r4}$	0.14	0.15	0.14	0.15	0.23	0.21	0.17	0.27
	$d_{r5}$	0.25	0.32	0.28	0.24	0.27	0.21	0.26	0.22
	$\alpha _r$	0.56	0.60	0.55	0.47	0.44	0.71	0.60	0.61
Linked by	$\theta _j$	0.60	0.34	0.43	0.70	0.45	0.36	0.41	0.50
five common examinees	$\beta _r$	0.46	0.23	0.34	0.68	0.30	0.21	0.34	0.37
	$d_{r2}$	0.69	0.35	0.33	0.82	0.45	0.44	0.41	0.53
	$d_{r3}$	0.36	0.24	0.24	0.44	0.30	0.31	0.26	0.31
	$d_{r4}$	0.29	0.18	0.18	0.33	0.24	0.22	0.20	0.28
	$d_{r5}$	0.72	0.43	0.38	0.91	0.35	0.37	0.39	0.37
	$\alpha _r$	0.81	0.66	0.60	0.81	0.57	0.80	1.04	0.79
Linked by	$\theta _j$	0.43	0.36	0.40	0.39	0.36	0.32	0.31	0.39
ten common examinees	$\beta _r$	0.31	0.24	0.30	0.32	0.19	0.20	0.20	0.27
	$d_{r2}$	0.38	0.43	0.33	0.30	0.40	0.40	0.36	0.45
	$d_{r3}$	0.24	0.28	0.21	0.24	0.29	0.28	0.27	0.35
	$d_{r4}$	0.22	0.22	0.19	0.18	0.21	0.21	0.19	0.28
	$d_{r5}$	0.39	0.51	0.35	0.37	0.28	0.25	0.33	0.33
	$\alpha _r$	0.59	0.69	0.58	0.56	0.53	0.68	0.65	0.69

**Table 4:** Means and SDs of the gold-standard parameters for GMFM

			Conditions							
			1	2	3	4	5	6	7	8
Reference	$\theta _j$	Mean	0.03	0.04	0.03	0.03	0.02	0.02	0.02	0.01
		SD	0.81	0.83	0.82	0.86	0.88	0.90	0.90	0.91
	$\beta _r$	Mean	$-1.12$	−1.14	−1.02	−0.91	−0.51	−0.53	−0.76	−0.39
		SD	0.48	0.48	0.45	0.38	0.50	0.48	0.45	0.46
	$d_{r2}$	Mean	−1.25	−1.16	−1.35	−1.19	−1.22	−1.14	−1.17	−1.22
		SD	0.44	0.38	0.42	0.40	0.36	0.37	0.34	0.34
	$d_{r3}$	Mean	−0.49	−0.46	−0.51	−0.50	−0.47	−0.43	−0.42	−0.44
		SD	0.39	0.32	0.39	0.44	0.34	0.32	0.25	0.25
	$d_{r4}$	Mean	0.41	0.39	0.44	0.40	0.39	0.38	0.39	0.41
		SD	0.22	0.16	0.23	0.21	0.19	0.18	0.20	0.19
	$d_{r5}$	Mean	1.34	1.23	1.42	1.29	1.30	1.19	1.19	1.24
		SD	0.53	0.44	0.47	0.48	0.41	0.42	0.33	0.34
	$\alpha _r$	Mean	1.55	1.67	1.49	1.66	1.65	1.67	1.71	1.73
		SD	0.69	0.62	0.60	0.75	0.69	0.62	0.67	0.70
Focal	$\theta _j$	Mean	−0.71	−0.73	−0.74	−0.73	0.62	0.37	−0.32	0.38
		SD	0.90	0.67	0.91	0.68	0.83	0.70	0.70	0.72
	$\beta _r$	Mean	−1.01	−0.87	−1.16	−1.12	−0.24	−0.37	−0.82	−0.52
		SD	0.44	0.31	0.47	0.45	0.35	0.32	0.35	0.36
	$d_{r2}$	Mean	−1.34	−1.22	−1.29	−1.21	−1.30	−1.18	−1.13	−1.14
		SD	0.38	0.39	0.43	0.37	0.44	0.37	0.40	0.41
	$d_{r3}$	Mean	−0.48	−0.43	−0.48	−0.41	−0.48	−0.44	−0.44	−0.45
		SD	0.37	0.37	0.39	0.25	0.40	0.36	0.42	0.42
	$d_{r4}$	Mean	0.45	0.40	0.43	0.39	0.44	0.39	0.37	0.36
		SD	0.20	0.22	0.20	0.18	0.22	0.19	0.17	0.19
	$d_{r5}$	Mean	1.37	1.26	1.34	1.22	1.33	1.23	1.21	1.22
		SD	0.42	0.44	0.50	0.40	0.51	0.43	0.50	0.52
	$\alpha _r$	Mean	1.58	1.74	1.59	1.72	1.57	1.83	1.82	1.72
		SD	0.63	0.82	0.68	0.66	0.67	0.84	0.81	0.76

*Note:* SD, standard deviation. All values are the averages of results obtained from ten experimental repetitions

**Table 5:** Means and SDs of parameters estimated solely from each group’s data

			Conditions							
			1	2	3	4	5	6	7	8
Reference	$\theta _j$	Mean	0.03	0.04	0.03	0.03	0.02	0.02	0.02	0.01
		SD	0.81	0.83	0.82	0.86	0.88	0.90	0.90	0.91
	$\beta _r$	Mean	−1.19	−1.21	−1.08	−0.96	−0.52	−0.57	−0.80	−0.42
		SD	0.46	0.45	0.41	0.37	0.54	0.51	0.45	0.49
	$d_{r2}$	Mean	−0.97	−0.91	−1.11	−0.91	−1.31	−1.24	−1.02	−1.32
		SD	0.54	0.42	0.53	0.42	0.44	0.44	0.40	0.40
	$d_{r3}$	Mean	−0.61	−0.47	−0.62	−0.54	−0.51	−0.43	−0.44	−0.41
		SD	0.42	0.44	0.46	0.46	0.35	0.35	0.30	0.29
	$d_{r4}$	Mean	0.25	0.22	0.34	0.22	0.44	0.46	0.31	0.51
		SD	0.39	0.29	0.36	0.30	0.27	0.27	0.27	0.29
	$d_{r5}$	Mean	1.33	1.15	1.39	1.22	1.38	1.20	1.15	1.22
		SD	0.53	0.47	0.47	0.48	0.42	0.42	0.35	0.34
	$\alpha _r$	Mean	1.25	1.34	1.27	1.34	1.44	1.49	1.49	1.55
		SD	0.47	0.40	0.46	0.55	0.57	0.48	0.54	0.53
Focal	$\theta _j$	Mean	0.01	0.00	0.01	0.01	0.03	0.03	0.02	0.03
		SD	0.89	0.82	0.89	0.83	0.84	0.82	0.84	0.82
	$\beta _r$	Mean	−0.26	−0.13	−0.38	−0.43	−0.90	−0.88	−0.59	−1.04
		SD	0.47	0.44	0.50	0.58	0.36	0.38	0.44	0.43
	$d_{r2}$	Mean	−1.39	−1.62	−1.33	−1.49	−1.13	−1.16	−1.39	−1.03
		SD	0.42	0.51	0.45	0.52	0.46	0.46	0.57	0.51
	$d_{r3}$	Mean	−0.52	−0.62	−0.50	−0.63	−0.60	−0.70	−0.63	−0.64
		SD	0.35	0.38	0.36	0.36	0.43	0.48	0.45	0.52
	$d_{r4}$	Mean	0.48	0.52	0.44	0.46	0.33	0.31	0.46	0.24
		SD	0.27	0.35	0.26	0.31	0.29	0.30	0.30	0.37
	$d_{r5}$	Mean	1.44	1.72	1.40	1.66	1.40	1.55	1.57	1.43
		SD	0.40	0.48	0.46	0.49	0.50	0.51	0.55	0.59
	$\alpha _r$	Mean	1.45	1.24	1.45	1.28	1.25	1.24	1.34	1.24
		SD	0.48	0.52	0.57	0.47	0.48	0.50	0.59	0.51

*Note:* SD, standard deviation. All values are the averages of results obtained from ten experimental repetitions

**Table 6:** Statistics of ability values predicted by the BERT-based AES model and linking coefficients calculated by the proposed method

		Conditions							
		1	2	3	4	5	6	7	8
$\theta _j$ predicted	Mean	−0.37	−0.48	−0.41	−0.54	0.54	0.44	−0.18	0.46
by BERT−AES	SD	0.75	0.69	0.72	0.66	0.70	0.70	0.74	0.72
	RMSE	0.61	0.53	0.62	0.49	0.45	0.42	0.46	0.41
Linking	A	0.85	0.84	0.82	0.80	0.83	0.85	0.88	0.88
coefficients	K	−0.38	−0.48	−0.42	−0.55	0.52	0.42	−0.19	0.43

*Note:* SD, standard deviation; RMSE, root mean squared error. All values are the averages of results obtained from ten experimental repetitions

The last three rows in Table 2 shows the results. We can see that the *PPP*-value for GMFM is close to 0.5, indicating a good fit to the data. In contrast, the other models exhibit high values, suggesting a poor fit to the data. Furthermore, among the three IRT models evaluated, GMFM exhibits the lowest WAIC and WBIC values. These findings suggest that GMFM offers the best fit to the data, corroborating previous work that investigated the same dataset using IRT models (Uto & Okano, 2021). We provide further discussion about the model fit in the *Analysis of rater characteristics* section given later.

According to these results, the following section focuses on the results for GMFM. Note that we also include the results for MFRM and MFRM with RSS in Appendix B, along with the open practices statement.

**Table 7:** Means and SDs of focal groups’ parameter estimates linked by the proposed method

		Conditions							
		1	2	3	4	5	6	7	8
$\theta _j$	Mean	−0.37	−0.48	−0.41	−0.54	0.54	0.44	−0.18	0.46
	SD	0.75	0.69	0.72	0.66	0.70	0.70	0.74	0.72
$\beta _r$	Mean	−0.59	−0.59	−0.73	−0.90	−0.23	−0.33	−0.71	−0.48
	SD	0.40	0.37	0.41	0.47	0.30	0.32	0.39	0.38
$d_{r2}$	Mean	−1.17	−1.36	−1.09	−1.19	−0.94	−0.99	−1.22	−0.90
	SD	0.36	0.43	0.37	0.42	0.39	0.40	0.50	0.45
$d_{r3}$	Mean	−0.44	−0.52	−0.41	−0.50	−0.50	−0.60	−0.56	−0.56
	SD	0.30	0.32	0.29	0.29	0.36	0.41	0.40	0.46
$d_{r4}$	Mean	0.40	0.44	0.36	0.37	0.28	0.26	0.40	0.21
	SD	0.23	0.30	0.21	0.24	0.24	0.26	0.26	0.32
$d_{r5}$	Mean	1.21	1.45	1.14	1.32	1.16	1.32	1.37	1.26
	SD	0.34	0.41	0.38	0.39	0.41	0.44	0.49	0.52
$\alpha _r$	Mean	1.74	1.48	1.79	1.61	1.51	1.47	1.54	1.42
	SD	0.57	0.62	0.70	0.60	0.58	0.60	0.68	0.58

*Note:* SD, standard deviation. All values are the averages of results obtained from ten experimental repetitions

**Table 8:** Absolute differences in the mean and SD values of the parameters for the focal groups between the proposed method and the gold-standard condition, as well as those between the unlinked condition and the gold-standard

			Conditions							
			1	2	3	4	5	6	7	8
Unlinked	$\theta _j$	Mean	0.72	0.74	0.75	0.74	0.59	0.34	0.34	0.35
		SD	0.03	0.15	0.03	0.15	0.02	0.12	0.14	0.10
	$\beta _r$	Mean	0.75	0.75	0.77	0.69	0.66	0.51	0.23	0.52
		SD	0.04	0.13	0.03	0.14	0.03	0.06	0.09	0.07
	$d_{r2}$	Mean	0.06	0.40	0.05	0.28	0.17	0.06	0.26	0.12
		SD	0.04	0.12	0.04	0.15	0.05	0.10	0.17	0.11
	$d_{r3}$	Mean	0.05	0.19	0.04	0.22	0.12	0.26	0.19	0.20
		SD	0.09	0.08	0.09	0.12	0.07	0.12	0.06	0.11
	$d_{r4}$	Mean	0.04	0.12	0.03	0.07	0.11	0.08	0.10	0.12
		SD	0.06	0.13	0.06	0.13	0.07	0.11	0.12	0.18
	$d_{r5}$	Mean	0.08	0.47	0.07	0.43	0.07	0.32	0.36	0.21
		SD	0.06	0.08	0.06	0.10	0.05	0.08	0.06	0.08
	$\alpha _r$	Mean	0.13	0.49	0.15	0.44	0.32	0.58	0.48	0.48
		SD	0.16	0.30	0.16	0.19	0.19	0.34	0.24	0.26
Linked by	$\theta _j$	Mean	0.34	0.25	0.33	0.19	0.10	0.11	0.14	0.08
proposed method		SD	0.14	0.04	0.18	0.05	0.13	0.03	0.05	0.02
	$\beta _r$	Mean	0.41	0.28	0.42	0.22	0.06	0.11	0.12	0.07
		SD	0.04	0.06	0.06	0.04	0.05	0.03	0.05	0.02
	$d_{r2}$	Mean	0.17	0.15	0.20	0.09	0.36	0.19	0.12	0.23
		SD	0.03	0.05	0.06	0.05	0.07	0.07	0.10	0.06
	$d_{r3}$	Mean	0.04	0.09	0.07	0.09	0.05	0.16	0.11	0.12
		SD	0.10	0.10	0.11	0.09	0.09	0.08	0.07	0.05
	$d_{r4}$	Mean	0.05	0.05	0.07	0.04	0.17	0.12	0.05	0.15
		SD	0.04	0.07	0.03	0.07	0.04	0.07	0.09	0.13
	$d_{r5}$	Mean	0.16	0.20	0.20	0.14	0.17	0.10	0.17	0.06
		SD	0.08	0.09	0.12	0.06	0.10	0.03	0.05	0.04
	$\alpha _r$	Mean	0.19	0.28	0.21	0.13	0.07	0.36	0.28	0.31
		SD	0.14	0.23	0.14	0.07	0.12	0.27	0.20	0.22

*Note:* SD, standard deviation. All values are the averages of results obtained from ten experimental repetitions

### Effectiveness of our proposed linking method

The results of the aforementioned experiments for GMFM are shown in Table 3. In the table, the *Unlinked* row represents the average RMSE between the focal group’s IRT parameters without applying our linking method and their gold-standard parameters. Similarly, the *Linked by proposed method* row represents the average RMSE between the focal group’s IRT parameters after applying our linking method and their gold-standard parameters. The rows labeled *Linked by five/ten common examinees* represent the results for linear linking using common examinees.

A comparison of the results from the unlinked condition and the proposed method reveals that the proposed method improved the RMSEs for the ability and rater severity parameters, namely, $\theta _j$ and $\beta _r$, which we intentionally varied between the reference and focal groups. The degree of improvement is notably substantial when the distributional differences between the reference and focal groups are large, as is the case in Conditions 1–5. On the other hand, for Conditions 6–8, where the distributional differences are relatively minor, the improvements are also smaller in comparison. This is because the RMSEs for the unlinked parameters are already lower in these conditions than in Conditions 1–5. Nonetheless, it is worth emphasizing that the RMSEs after employing our linking method are exceptionally low in Conditions 6–8.

Furthermore, the table indicates that the RMSEs for the step parameters and rater consistency parameters, namely, $d_{rm}$ and $\alpha _r$, also improved in many cases, while the impact of applying our linking method is relatively small for these parameters compared with the ability and rater severity parameters. This is because we did not intentionally vary their distribution between the reference and focal groups, and thus their distribution differences were smaller than those for the ability and rater severity parameters, as shown in the next section.

Comparing the results from the proposed method and linear linking using five common examinees, we observe that the proposed method generally exhibits lower RMSE values for the ability $\theta _j$ and the rater severity parameters $\beta _r$, except for conditions 2–3. Furthermore, when comparing the proposed method with linear linking using ten common examinees, it achieves superior performance in conditions 4–8 and slightly lower performance in conditions 1–3 for $\theta _j$ and $\beta _r$, while the differences are more minor overall than those observed when comparing the proposed method with the condition of five common examinees. Note that the reasons why the proposed method tends to show lower performance for conditions 1–3 are as follows.

1. The proposed method utilizes fewer samples to estimate the rater parameters compared with the linear linking method using common examinees.
2. In situations where distributional differences between the reference and focal groups are relatively large, as in conditions 1–3, constructing an accurate AES model for the focal group becomes challenging due to the limited overlap in the ability value range. We elaborate on this point in the next section.

Furthermore, in terms of the rater consistency parameter $\alpha _r$ and the step parameter $d_{rm}$, the proposed method typically shows lower RMSE values compared with linear linking using common examinees. We attribute this to the fact that the performance of the linking method using common examinees is highly dependent on the choice of common examinees, which can sometimes result in significant errors in these parameters. This issue is also further discussed in the next section.

These results suggest that our method can perform linking with comparable accuracy to linear linking using few common examinees, even in the absence of common examinees and raters. Additionally, as reported in Tables 15 and 16 in Appendix B, both MFRM and MFRM with RSS also exhibit a similar tendency, further validating the effectiveness of our approach regardless of the IRT models employed.

### Detailed analysis

#### Analysis of parameter scale transformation using the proposed method

In this section, we detail how our method transforms the parameter scale. To demonstrate this, we first summarize the mean and SD values of the gold-standard parameters for both the reference and focal groups in Table 4. The values in the table are averages calculated from ten repetitions of the experimental procedures. The table shows that the mean and SD values of both examinee ability and rater severity vary significantly between the reference and focal groups following our intended settings, as outlined in Table 1. Additionally, the mean and SD values for the rater consistency parameter $\alpha _r$ and the rater-specific step parameters $d_{rm}$ also differ slightly between the groups, although we did not intentionally alter them.

Second, the averaged values of the means and SDs of the parameters, estimated solely from either the reference or the focal group’s data over ten repetitions, are presented in Table 5. The table reveals that the estimated parameters for both groups align with a normal distribution centered at nearly zero, despite the actual ability distributions differing between the groups. This phenomenon arises because IRT permits arbitrary scaling of parameters for each independent dataset, as mentioned in the *Linking* section. This leads to differences in the parameter scale for the focal group compared with their gold-standard values, thereby highlighting the need for parameter linking.

Next, the first two rows of Table 6 display the mean and SD values of the ability estimates for the focal group’s examinees, as predicted by the BERT-based AES model. In the table, the *RMSE* row indicates the RMSE between the AES-predicted ability values and the gold-standard ability values for the focal groups. The *Linking Coefficients* row presents the linking coefficients calculated based on the AES-predicted abilities. As with the abovementioned tables, these values are also averages over ten experimental repetitions. According to the table, for Conditions 6–8, where the distributional differences between the groups are relatively minor, both the mean and SD estimates align closely with those of the gold-standard parameters. In contrast, for Conditions 1–5, where the distributional differences are more pronounced, the mean and SD estimates tend to deviate from the gold-standard values, highlighting the challenges of parameter linking under such conditions.

In addition, as indicated in the RMSE row, the AES-predicted abilities may lack accuracy under specific conditions, such as Conditions 1, 2, and 3. This inaccuracy could arise because the AES model, trained on the reference group’s data, could not cover the ability range of the focal group due to significant differences in the ability distribution between the groups. Note that even in cases where the mean and SD estimates are relatively inaccurate, these values are closer to the gold-standard ones than those estimated solely from the focal group’s data. This leads to meaningful linking coefficients, which transform the focal group’s parameters toward the scale of their gold-standard values.

Finally, Table 7 displays the averaged values of the means and SDs of the focal group’s parameters obtained through our linking method over ten repetitions. Note that the mean and SD values of the ability estimates are the same as those reported in Table 6 because the proposed method is designed to align them. The table indicates that the differences in the mean and SD values between the proposed method and the gold-standard condition, shown in Table 4, tend to be smaller compared with those between the unlinked condition, shown in Table 5, and the gold-standard. To verify this point more precisely, Table 8 shows the average absolute differences in the mean and SD values of the parameters for the focal groups between the proposed method and the gold-standard condition, as well as those between the unlinked condition and the gold-standard. These values were calculated by averaging the absolute differences in the mean and SD values obtained from each of the ten repetitions, unlike the simple absolute differences in the values reported in Tables 4 and 7. The table shows that the proposed linking method tends to derive lower values, especially for $\theta _j$ and $\beta _r$, than the unlinked condition. Furthermore, this tendency is prominent for conditions 6–8 in which the distributional differences between the focal and reference groups are relatively small. These trends are consistent with the cases for which our method revealed high linking performance, detailed in the previous section.

In summary, the above analyses suggest that although the AES model’s predictions may not always be perfectly accurate, they can offer valuable insights into scale differences between the reference and focal groups, thereby facilitating successful IRT parameter linking without common examinees and raters.

**Fig. 5:**
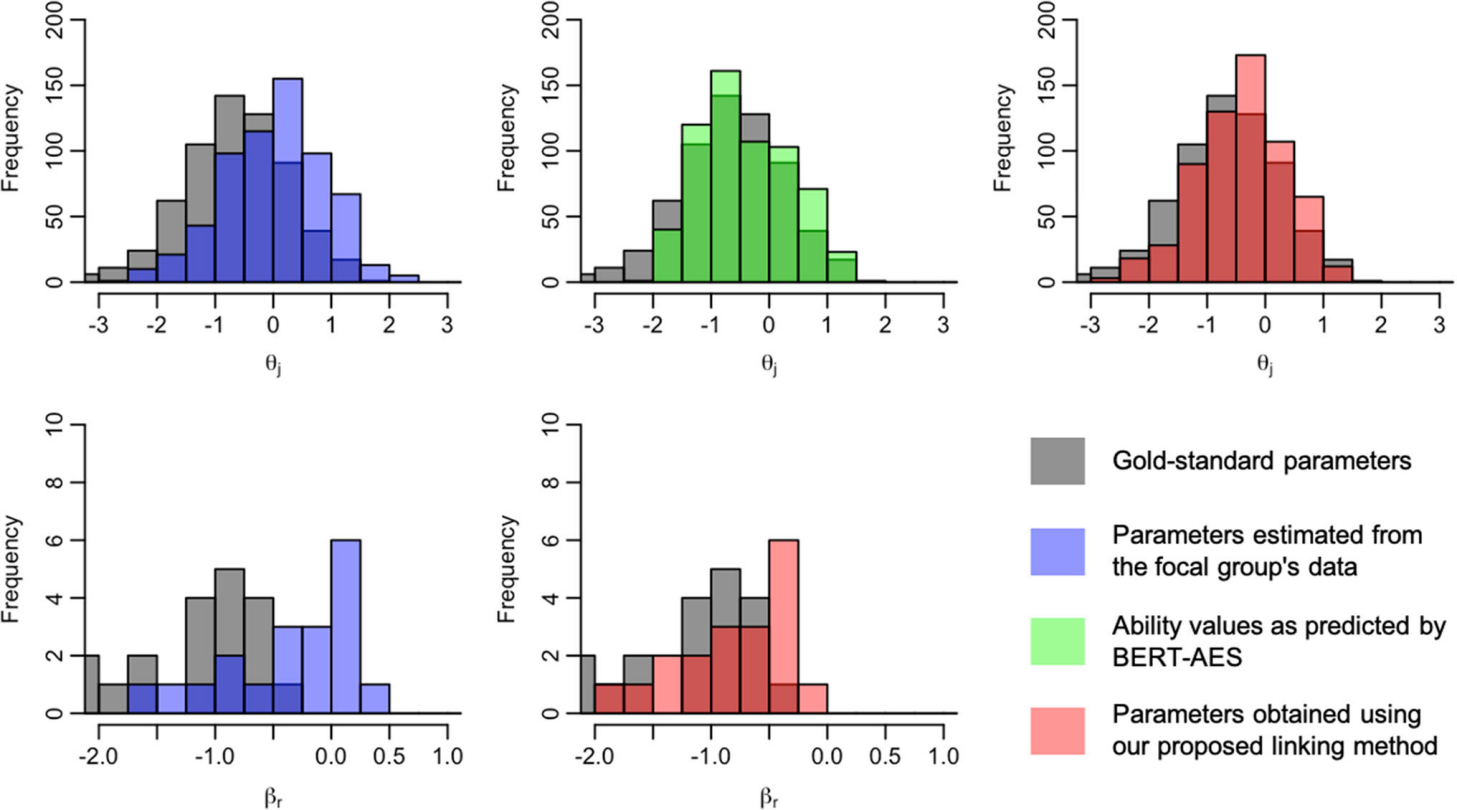
Example of ability and rater severity distributions for the focal group under data-splitting condition 1

**Fig. 6:**
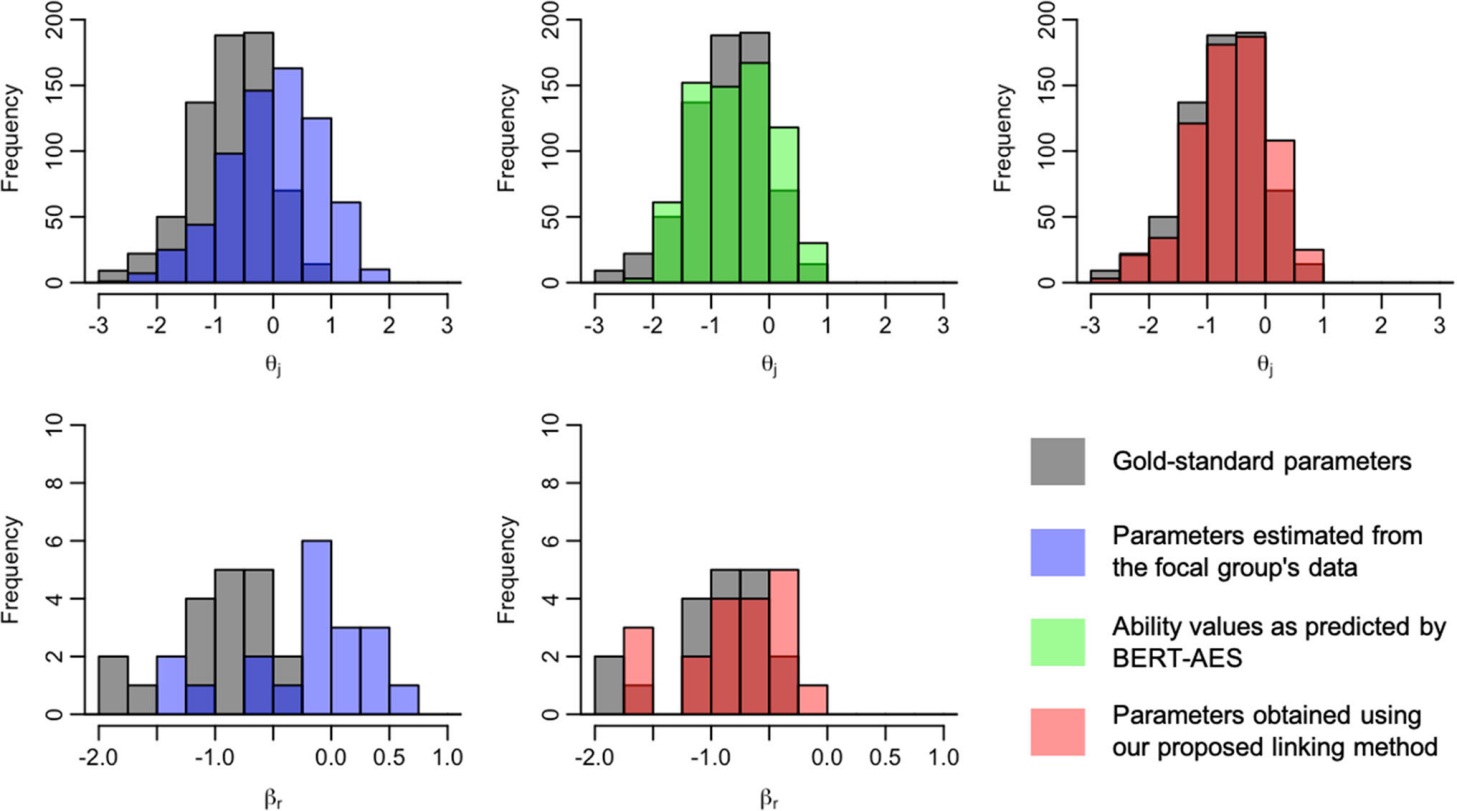
Example of ability and rater severity distributions for the focal group under data-splitting condition 2

**Fig. 7:**
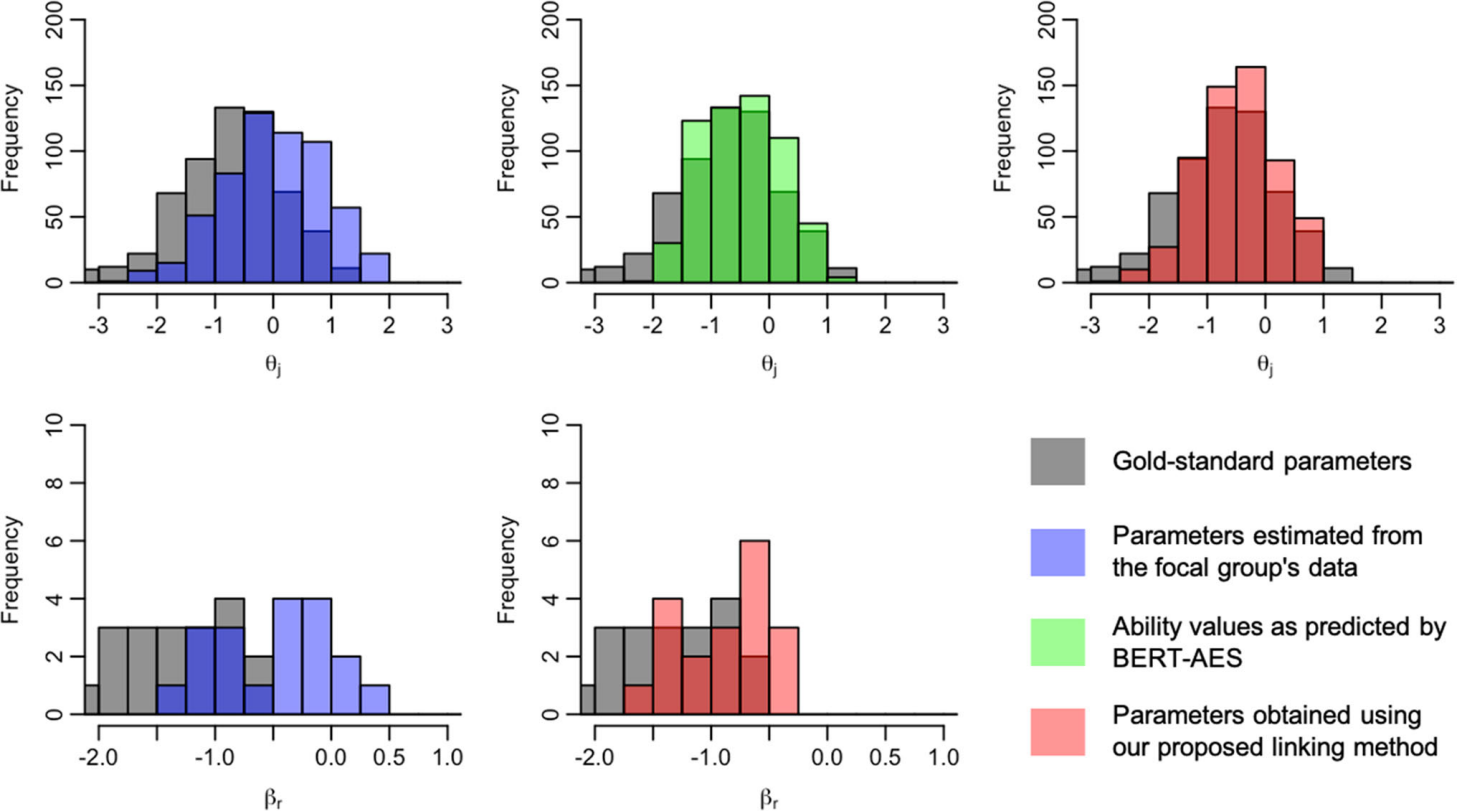
Example of ability and rater severity distributions for the focal group under data-splitting condition 3

**Fig. 8:**
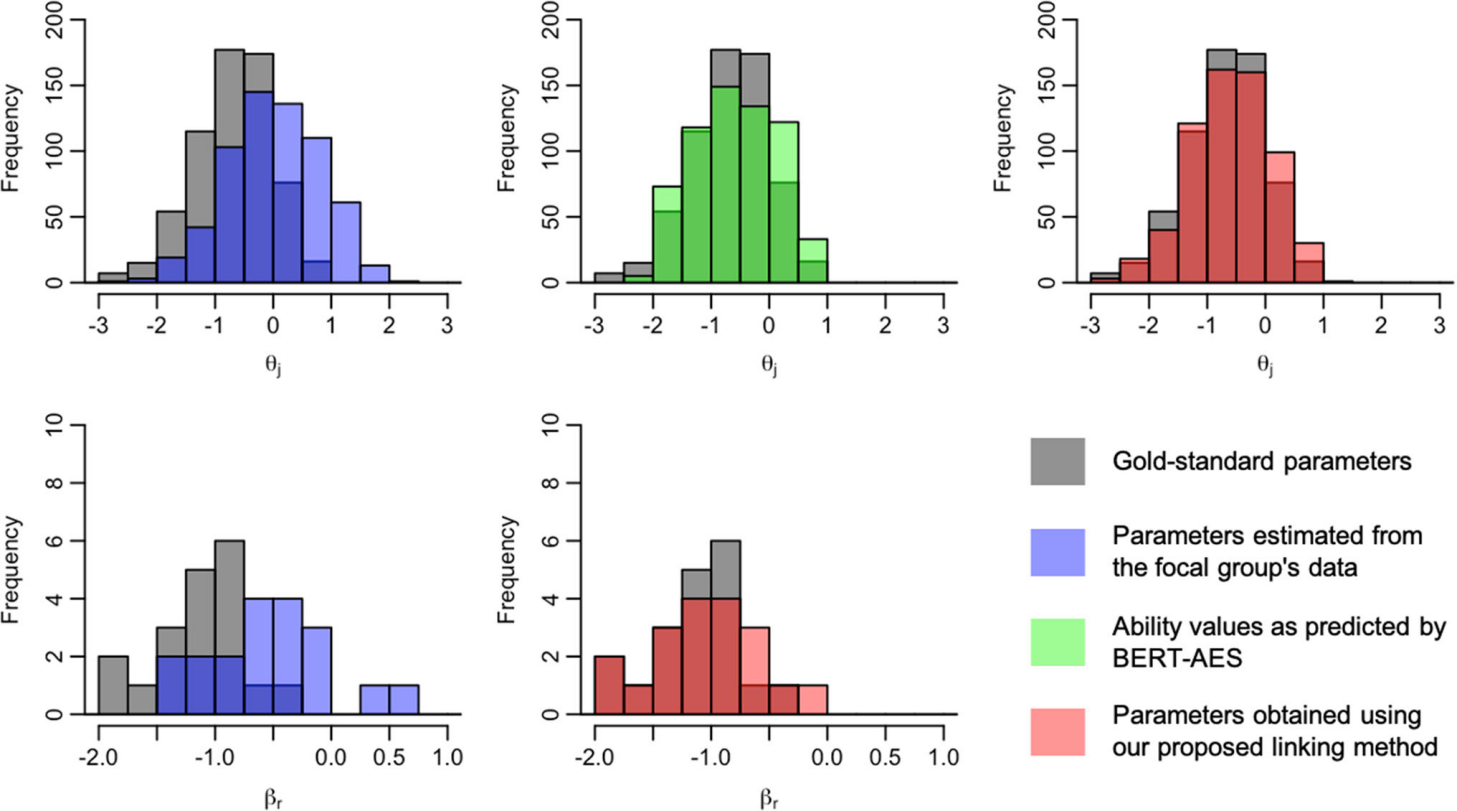
Example of ability and rater severity distributions for the focal group under data-splitting condition 4

**Fig. 9:**
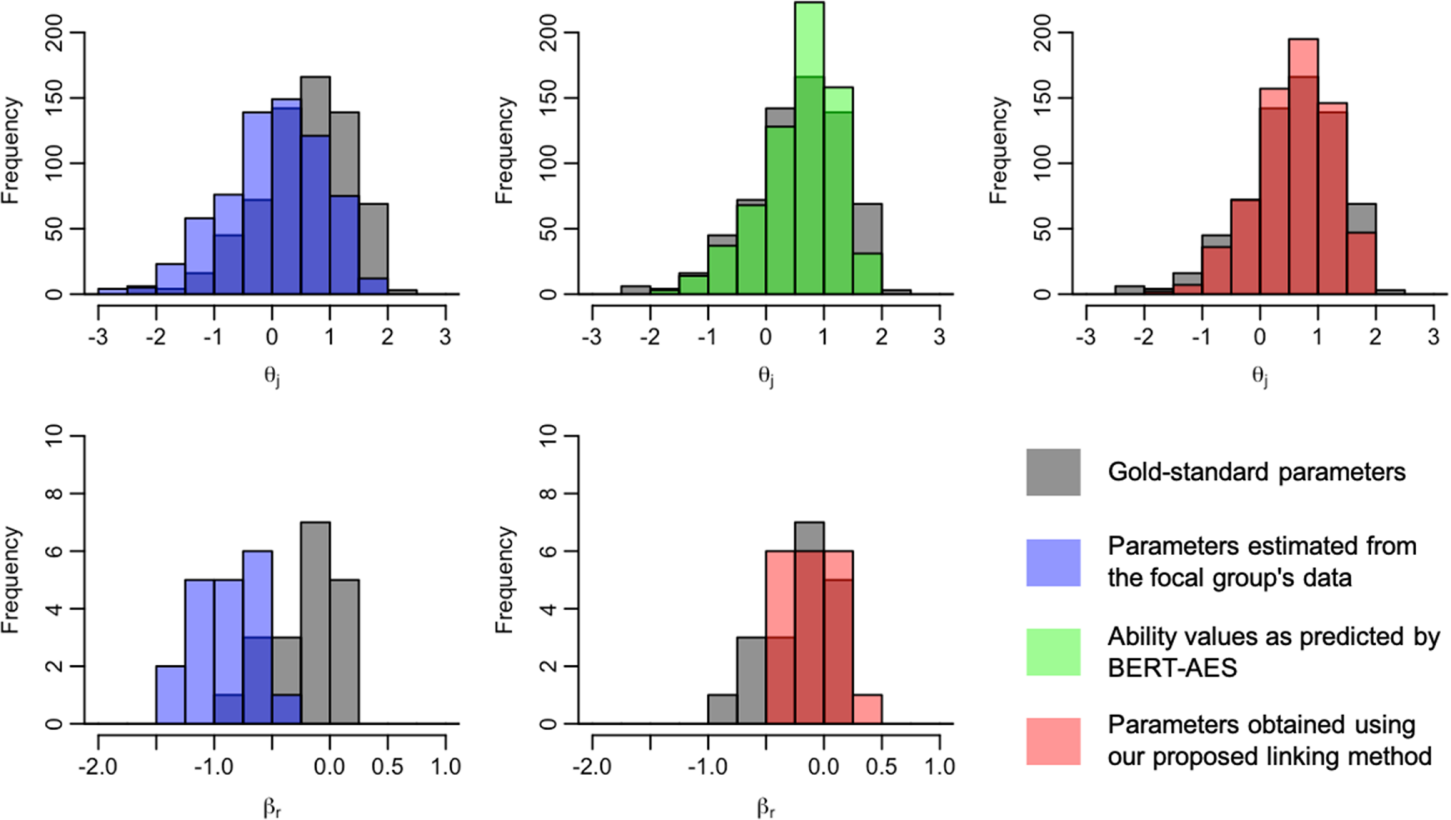
Example of ability and rater severity distributions for the focal group under data-splitting condition 5

**Fig. 10:**
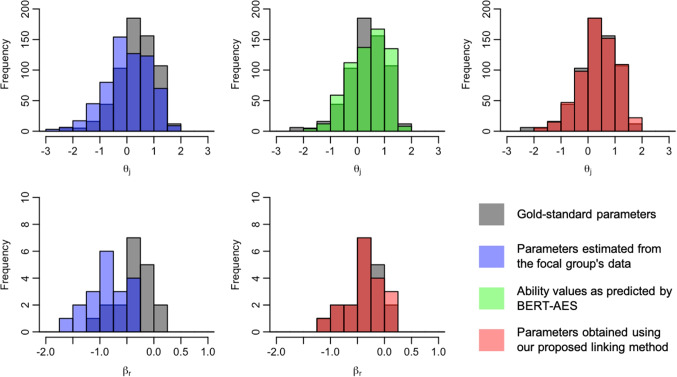
Example of ability and rater severity distributions for the focal group under data-splitting condition 6

**Fig. 11:**
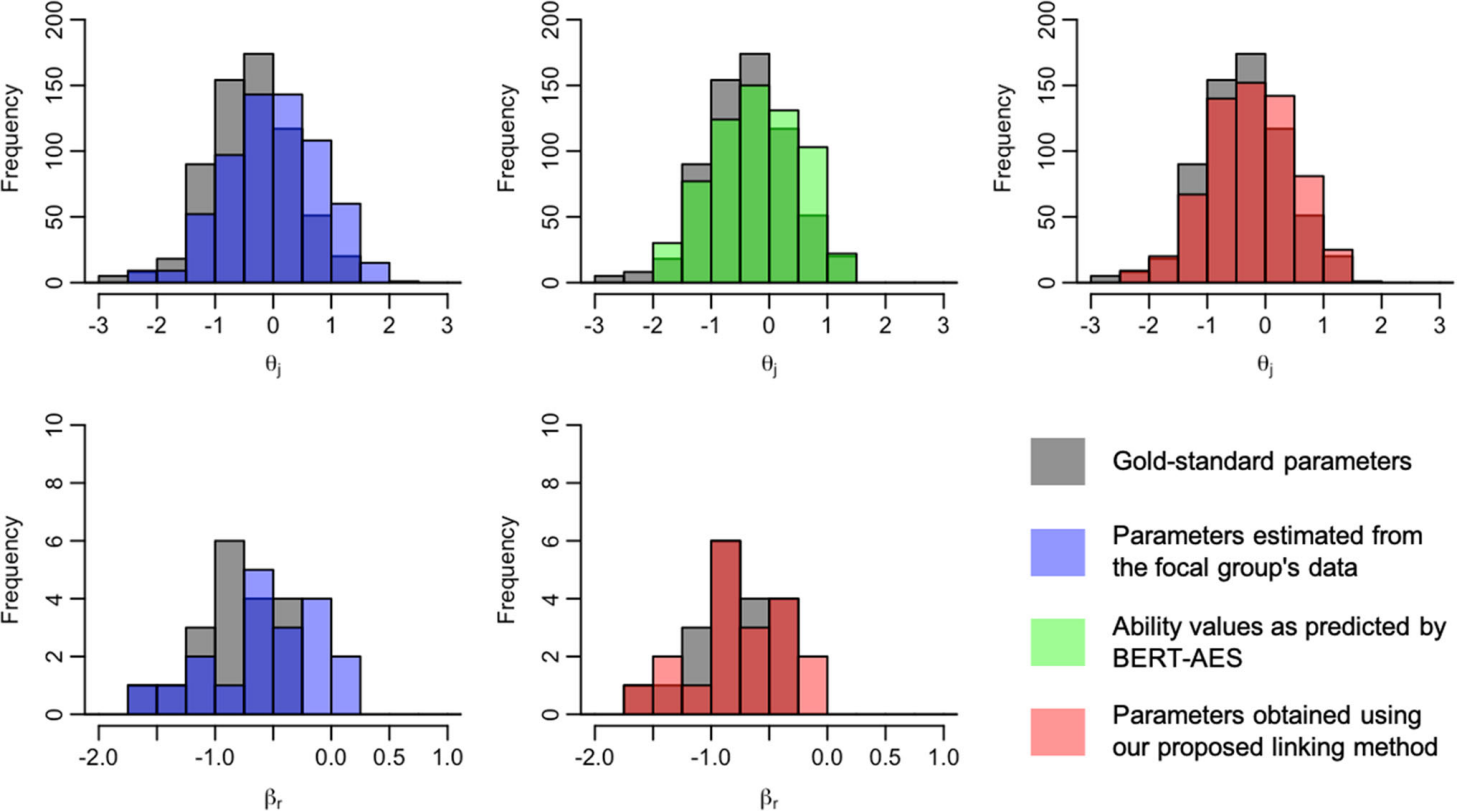
Example of ability and rater severity distributions for the focal group under data-splitting condition 7

**Fig. 12:**
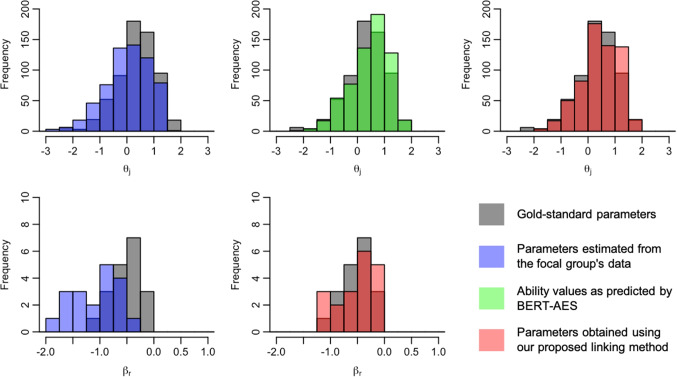
Example of ability and rater severity distributions for the focal group under data-splitting condition 8

We now present the distributions of examinee ability and rater severity for the focal group, comparing their gold-standard values with those before and after the application of the linking method. Figures 5, 6, 7, 8, 9, 10, 11, and 12 are illustrative examples for the eight data-splitting conditions. The gray bars depict the distributions of the gold-standard parameters, the blue bars represent those of the parameters estimated from the focal group’s data, the red bars signify those of the parameters obtained using our linking method, and the green bars indicate the ability distribution as predicted by the BERT-based AES. The upper part of the figure presents results for examinee ability $\theta _j$ and the lower part presents those for rater severity $\beta _r$.

The blue bars in these figures reveal that the parameters estimated from the focal group’s data exhibit distributions with different locations and/or scales compared with their gold-standard values. Meanwhile, the red bars reveal that the distributions of the parameters obtained through our linking method tend to align closely with those of the gold-standard parameters. This is attributed to the fact that the ability distributions for the focal group given by the BERT-based AES model, as depicted by the green bars, were informative for performing linear linking.

#### Analysis of the linking method based on common examinees

For a detailed analysis of the linking method based on common examinees, Table 9 reports the averaged values of means and SDs of the focal groups’ parameter estimates obtained by the linking method based on five and ten common examinees for each condition. Furthermore, Table 10 shows the average absolute differences between these values and those from the gold standard condition. Table 10 shows that an increase in the number of common examinees tends to lower the average absolute differences, which is a reasonable trend. Furthermore, comparing the results with those of the proposed method reported in Table 8, the proposed method tends to achieve smaller absolute differences in conditions 4–8 for $\theta _j$ and $\beta _r$, which is consistent with the tendency of the linking performance discussed in the “Effectiveness of our proposed linking method” section.

Note that although the mean and SD values in Table 9 are close to those of the gold-standard parameters shown in Table 4, this does not imply that linear linking based on five or ten common examinees achieves high linking accuracy for each repetition. To explain this, Table 11 shows the means of the gold-standard ability values for the focal group and their estimates obtained from the proposed method and the linking method based on ten common examinees, for each of ten repetitions under condition 8. This table also shows the absolute differences between the estimated ability means and the corresponding gold-standard means.

**Table 9:** Means and SDs of focal groups’ parameter estimates linked by the linear linking method based on five and ten common examinees

			Conditions							
			1	2	3	4	5	6	7	8
Linked by	$\theta _j$	Mean	$-0.63$	$-0.65$	$-0.59$	$-0.92$	0.55	0.31	$-0.13$	0.05
five common		SD	1.09	0.73	0.89	0.97	0.82	0.69	0.65	0.62
examinees	$\beta _r$	Mean	$-0.95$	$-0.77$	$-1.00$	$-1.43$	$-0.36$	$-0.45$	$-0.60$	$-0.77$
		SD	0.58	0.39	0.50	0.67	0.36	0.32	0.34	0.33
	$d_{r2}$	Mean	$-1.69$	$-1.44$	$-1.34$	$-1.77$	$-1.10$	$-0.97$	$-1.08$	$-0.77$
		SD	0.52	0.45	0.46	0.62	0.46	0.38	0.44	0.40
	$d_{r3}$	Mean	$-0.64$	$-0.56$	$-0.52$	$-0.74$	$-0.60$	$-0.59$	$-0.49$	$-0.50$
		SD	0.44	0.35	0.37	0.42	0.43	0.40	0.37	0.40
	$d_{r4}$	Mean	0.57	0.46	0.44	0.55	0.33	0.26	0.36	0.18
		SD	0.32	0.31	0.25	0.34	0.28	0.25	0.23	0.28
	$d_{r5}$	Mean	1.75	1.54	1.41	1.96	1.37	1.30	1.21	1.09
		SD	0.49	0.43	0.47	0.57	0.49	0.43	0.44	0.45
	$\alpha _r$	Mean	1.43	1.46	1.55	1.31	1.38	1.60	2.09	1.81
		SD	0.48	0.62	0.61	0.47	0.53	0.65	0.93	0.75
Linked by	$\theta _j$	Mean	$-0.84$	$-0.74$	$-0.69$	$-0.54$	0.62	0.33	$-0.34$	0.33
ten common		SD	1.02	0.80	0.94	0.75	0.81	0.67	0.76	0.75
examinees	$\beta _r$	Mean	$-1.14$	$-0.86$	$-1.12$	$-0.95$	$-0.27$	$-0.41$	$-0.89$	$-0.65$
		SD	0.54	0.42	0.52	0.52	0.35	0.31	0.40	0.39
	$d_{r2}$	Mean	$-1.59$	$-1.58$	$-1.42$	$-1.35$	$-1.09$	$-0.96$	$-1.26$	$-0.95$
		SD	0.47	0.50	0.47	0.47	0.44	0.38	0.52	0.46
	$d_{r3}$	Mean	$-0.59$	$-0.61$	$-0.53$	$-0.56$	$-0.58$	$-0.57$	$-0.58$	$-0.58$
		SD	0.40	0.36	0.39	0.33	0.40	0.40	0.42	0.48
	$d_{r4}$	Mean	0.54	0.51	0.46	0.41	0.33	0.26	0.42	0.22
		SD	0.30	0.34	0.27	0.28	0.27	0.25	0.27	0.33
	$d_{r5}$	Mean	1.64	1.68	1.49	1.49	1.35	1.27	1.42	1.31
		SD	0.45	0.47	0.49	0.44	0.47	0.42	0.51	0.54
	$\alpha _r$	Mean	1.35	1.31	1.43	1.45	1.34	1.56	1.54	1.41
		SD	0.45	0.55	0.56	0.53	0.53	0.63	0.68	0.59

*Note:* SD, standard deviation. All values are the averages of results obtained from ten experimental repetitions

**Table 10:** Absolute differences in the mean and SD values of the parameters for the focal groups between the linear linking method based on common examinees and the gold-standard condition

			Conditions							
			1	2	3	4	5	6	7	8
Linked by	$\theta _j$	Mean	0.30	0.16	0.23	0.45	0.29	0.17	0.26	0.39
five common examinees		SD	0.39	0.12	0.19	0.37	0.16	0.16	0.18	0.15
	$\beta _r$	Mean	0.36	0.17	0.30	0.61	0.25	0.15	0.30	0.32
		SD	0.22	0.10	0.10	0.27	0.07	0.09	0.09	0.08
	$d_{r2}$	Mean	0.64	0.28	0.27	0.72	0.28	0.27	0.30	0.37
		SD	0.20	0.09	0.10	0.27	0.11	0.10	0.11	0.10
	$d_{r3}$	Mean	0.24	0.14	0.13	0.34	0.17	0.18	0.12	0.12
		SD	0.18	0.06	0.12	0.18	0.09	0.08	0.11	0.10
	$d_{r4}$	Mean	0.21	0.08	0.10	0.21	0.15	0.13	0.11	0.18
		SD	0.14	0.09	0.08	0.18	0.08	0.07	0.08	0.11
	$d_{r5}$	Mean	0.66	0.34	0.30	0.85	0.26	0.30	0.30	0.25
		SD	0.17	0.06	0.12	0.18	0.10	0.10	0.12	0.11
	$\alpha _r$	Mean	0.60	0.38	0.31	0.64	0.33	0.41	0.70	0.45
		SD	0.25	0.26	0.18	0.28	0.19	0.26	0.43	0.27
Linked by	$\theta _j$	Mean	0.23	0.18	0.19	0.26	0.18	0.18	0.11	0.21
ten common examinees		SD	0.17	0.14	0.16	0.10	0.09	0.08	0.11	0.13
	$\beta _r$	Mean	0.27	0.18	0.25	0.27	0.13	0.15	0.15	0.22
		SD	0.11	0.11	0.09	0.09	0.03	0.05	0.07	0.07
	$d_{r2}$	Mean	0.32	0.37	0.27	0.17	0.21	0.25	0.20	0.24
		SD	0.09	0.10	0.08	0.10	0.08	0.09	0.13	0.11
	$d_{r3}$	Mean	0.12	0.18	0.09	0.16	0.14	0.13	0.14	0.15
		SD	0.10	0.09	0.08	0.09	0.10	0.09	0.10	0.12
	$d_{r4}$	Mean	0.12	0.12	0.09	0.05	0.12	0.13	0.08	0.16
		SD	0.10	0.11	0.09	0.11	0.07	0.06	0.10	0.14
	$d_{r5}$	Mean	0.30	0.42	0.27	0.30	0.16	0.15	0.24	0.21
		SD	0.07	0.09	0.10	0.07	0.09	0.05	0.08	0.12
	$\alpha _r$	Mean	0.31	0.43	0.32	0.29	0.28	0.28	0.31	0.37
		SD	0.21	0.27	0.22	0.14	0.15	0.23	0.23	0.26

*Note:* SD, standard deviation. All values are the averages of results obtained from ten experimental repetitions

**Table 11:** Means of the gold-standard ability values for the focal group and their estimates obtained from each linking method for each of the ten repetitions under condition 8 as well as their absolute differences

	Ability means			Absolute differences	
Repetitions	Gold-standard	Proposed	CE10	|Gold - Prop|	|Gold - CE10|
1	0.33	0.44	0.02	0.11	0.31
2	0.42	0.57	0.44	0.15	0.02
3	0.34	0.43	0.55	0.09	0.21
4	0.33	0.31	$-0.14$	0.02	0.46
5	0.35	0.35	0.38	0.00	0.03
6	0.50	0.64	$-0.01$	0.14	0.51
7	0.42	0.52	0.40	0.10	0.02
8	0.35	0.43	0.40	0.08	0.05
9	0.37	0.40	0.48	0.03	0.11
10	0.42	0.46	0.76	0.04	0.34
Avg.	0.38	0.46	0.33	0.08	0.21

*Note:* CE10 represents the linear linking method based on the ten common examinees. The |Gold - Prop| column shows the absolute differences between the “Gold-standard” and “Proposed” columns. The |Gold - CE10| column shows the absolute differences between the “Gold-standard” and “CE10” columns. The last row shows the average of these differences over ten repetitions

**Table 12:** SD of RMSEs for ten repetitions

		Conditions							
		1	2	3	4	5	6	7	8
Linked by	$\theta _j$	0.06	0.07	0.08	0.06	0.03	0.06	0.06	0.02
proposed method	$\beta _r$	0.07	0.10	0.11	0.09	0.02	0.09	0.06	0.02
	$d_{r2}$	0.08	0.07	0.10	0.04	0.04	0.05	0.07	0.06
	$d_{r3}$	0.07	0.04	0.10	0.05	0.06	0.05	0.05	0.05
	$d_{r4}$	0.03	0.04	0.04	0.01	0.04	0.04	0.03	0.07
	$d_{r5}$	0.08	0.04	0.12	0.06	0.04	0.06	0.08	0.03
	$\alpha _r$	0.12	0.15	0.23	0.07	0.08	0.17	0.14	0.17
Linked by	$\theta _j$	0.23	0.19	0.10	0.07	0.12	0.07	0.11	0.12
ten common examinees	$\beta _r$	0.25	0.14	0.14	0.10	0.07	0.08	0.17	0.20
	$d_{r2}$	0.33	0.34	0.18	0.14	0.07	0.08	0.21	0.06
	$d_{r3}$	0.12	0.13	0.08	0.09	0.06	0.07	0.13	0.07
	$d_{r4}$	0.13	0.14	0.05	0.06	0.05	0.04	0.08	0.06
	$d_{r5}$	0.32	0.34	0.18	0.18	0.12	0.11	0.26	0.11
	$\alpha _r$	0.15	0.24	0.20	0.13	0.10	0.21	0.17	0.18

The table shows that the results of the proposed method are relatively stable, consistently revealing low absolute differences for every repetition. In contrast, the results of linear linking based on ten common examinees vary significantly across repetitions, resulting in large absolute differences for some repetitions. These results yield a smaller average absolute difference for the proposed method compared with linear linking based on ten common examinees. However, in terms of the absolute difference in the averaged ability means, linear linking based on ten common examinees shows a smaller difference ($|0.38-0.33| = 0.05$) compared with the proposed method ($|0.38-0.46| = 0.08$). This occurs because the results of linear linking based on ten common examinees for ten repetitions fluctuate around the ten-repetition average of the gold standard, thereby canceling out the positive and negative differences. However, this does not imply that linear linking based on ten common examinees achieves high linking accuracy for each repetition. Thus, it is reasonable to interpret the average of the absolute differences calculated for each of the ten repetitions, as reported in Tables 8 and 10.

This greater variability in performance of the linking method based on common examinees also relates to the tendency of the proposed method to show lower RMSE values for the rater consistency parameter $\alpha _r$ and the step parameters $d_{rm}$ compared with linking based on common examinees, as mentioned in the *Effectiveness of our proposed linking method* section. In that section, we mentioned that this is due to the fact that linear linking based on common examinees is highly dependent on the selection of common examinees, which can sometimes lead to significant errors in these parameters.

To confirm this point, Table 12 displays the SD of RMSEs calculated from ten repetitions of the experimental procedures for both the proposed method and linear linking using ten common examinees. The table indicates that the linking method using common examinees tends to exhibit larger SD values overall, suggesting that this linking method sometimes becomes inaccurate, as we also exemplified in Table 11. This variability also implies that the estimation of the linking coefficient can be unstable.

Furthermore, the tendency of having larger SD values in the common examinee approach is particularly pronounced for the step parameters at the extreme categories, namely, $d_{r2}$ and $d_{r5}$. We consider this comes from the instability of linking coefficients and the fact that the step parameters for the extreme categories tend to have large absolute values (see Table 13 for detailed estimates). Linear linking multiplies the step parameters by a linking coefficient *A*, although applying an inappropriate linking coefficient to larger absolute values can have a more substantial impact than when applied to smaller values. We concluded that this is why the RMSEs of the step difficulty parameters in the common examinee approach were deteriorated compared with those in the proposed method. The same reasoning would be applicable to the rater consistency parameter, given that it is distributed among positive values with a mean over one. See Table 13 for details.

## Discussion

### Prerequisites of the proposed method

As demonstrated thus far, the proposed method can perform IRT parameter linking without the need for common examinees and raters. As outlined in the *Introduction* section, certain testing scenarios may encounter challenges or incur significant costs in assembling common examinees or raters. Our method provides a viable solution in these situations. However, it does come with specific prerequisites and inherent costs.

**Table 13:** GMFM-based rater parameters calculated from actual data

Raters	$\alpha _r$	$\beta _r$	$d_{r2}$	$d_{r3}$	$d_{r4}$	$d_{r5}$
1	1.99	$-0.27$	$-1.53$	$-0.27$	0.48	1.32
2	0.87	$-0.15$	$-1.16$	$-0.69$	0.30	1.55
3	1.80	$-0.44$	$-1.03$	$-0.66$	0.51	1.18
4	1.13	$-0.54$	$-1.39$	$-0.50$	0.58	1.31
5	0.49	$-0.68$	$-0.60$	$-2.56$	0.22	2.94
6	2.19	$-1.21$	$-1.21$	$-0.24$	0.14	1.30
7	1.26	$-1.18$	$-1.46$	$-0.09$	0.26	1.30
8	1.33	$-1.54$	$-0.84$	$-0.44$	0.28	1.00
9	2.41	$-0.37$	$-1.76$	$-0.42$	0.63	1.54
10	1.72	$-1.80$	$-1.28$	$-0.50$	0.41	1.37
11	1.50	$-0.45$	$-1.32$	$-0.49$	0.58	1.23
12	1.15	$-1.26$	$-0.90$	$-0.71$	0.39	1.22
13	1.32	$-0.25$	$-1.88$	$-0.50$	0.84	1.55
14	2.05	$-0.41$	$-1.34$	$-0.50$	0.20	1.65
15	1.34	$-0.82$	$-1.28$	$-0.13$	0.26	1.15
16	0.65	$-0.89$	0.10	$-0.07$	0.52	$-0.55$
17	1.31	$-0.73$	$-1.69$	$-0.59$	0.54	1.74
18	2.19	$-0.24$	$-1.50$	$-0.41$	0.50	1.41
19	2.31	$-0.04$	$-1.75$	$-0.54$	0.67	1.63
20	2.41	$-0.71$	$-1.55$	$-0.32$	0.55	1.33
21	2.32	$-0.63$	$-1.26$	$-0.23$	0.41	1.07
22	3.89	$-0.46$	$-1.08$	$-0.25$	0.26	1.07
23	2.16	$-0.97$	$-1.39$	$-0.54$	0.46	1.47
24	1.09	$-0.75$	$-2.00$	$-0.67$	0.50	2.16
25	1.31	$-1.45$	$-1.01$	$-0.45$	0.41	1.05
26	2.47	$-0.54$	$-1.18$	$-0.34$	0.33	1.18
27	1.09	$-0.16$	$-1.42$	$-0.28$	0.53	1.17
28	1.61	$-0.82$	$-1.19$	$-0.75$	0.54	1.40
29	0.83	$-0.25$	$-1.03$	$-0.54$	0.24	1.33
30	1.09	$-0.51$	$-2.09$	$-0.70$	0.71	2.08
31	1.36	$-1.70$	$-1.34$	$-0.49$	0.18	1.65
32	1.57	$-0.52$	$-1.89$	$-0.37$	0.68	1.59
33	1.34	$-0.53$	$-1.34$	$-0.42$	0.42	1.35
34	0.59	$-0.26$	$-0.51$	0.01	$-0.31$	0.81
35	1.42	$-0.86$	$-1.63$	$-0.15$	0.67	1.11
36	1.50	$-0.89$	$-1.33$	$-0.39$	0.30	1.43
37	0.91	$-0.13$	$-1.43$	$-0.28$	0.81	0.90
38	1.27	$-1.47$	$-0.96$	$-1.10$	0.39	1.67
Avg.	1.56	$-0.71$	$-1.30$	$-0.49$	0.43	1.36

The prerequisites of our proposed method are as follows.

1. The same essay writing task is offered to both the reference and focal groups, and the written essays for it are scored by different groups of raters using the same rubric.
2. Raters will function identically across both the reference and focal groups, and the established scales can be adjusted through linear transformations. This implies that there are no systematic differences in scoring that are correlated with the groups but are unrelated to the measured construct, such as differential rater functioning (Leckie & Baird, 2011; Myford & Wolfe, 2009; Uto, 2023; Wind & Guo, 2019).
3. The ability ranges of the reference and focal groups require some overlap because the ability prediction accuracy of the AES decreases as the differences in the ability distributions between the groups increases, as discussed in the *Detailed analysis* section. This is a limitation of this approach, which requires future studies to overcome.
4. The reference group consists of a sufficient number of examinees for training AES models using their essays as training data.

Related to the fourth point, we conducted an additional experiment to investigate the number of samples required to train AES models. In this experiment, we assessed the ability prediction accuracy of the BERT-based AES model used in this study by varying the number of training samples. The detailed experimental procedures are outlined below.

1. Estimate the ability of all 1805 examinees from the entire dataset based on the GMFM.
2. Randomly split the examinees into 80% (1444) and 20% (361) groups. The 20% subset, consisting of examinees’ essays and their ability estimates, was used as test data to evaluate the ability prediction accuracy of the AES model trained through the following steps.
3. The 80% subset was further divided into 80% (1155) and 20% (289) groups. Here, the essays and ability estimates of the 80% subset were used as the training data, while those of the 20% served as development data for selecting the optimal epoch.
4. Train the BERT-based AES model using the training data and select the optimal epoch that minimizes the RMSE between the predicted and gold-standard ability values for the development set.
5. Use the trained AES model at the optimal epoch to evaluate the RMSE between the predicted and gold-standard ability values for the test data.
6. Randomly sample 50, 100, 200, 300, 500, 750, and 1000 examinees from the training data created in Step 3.
7. Train the AES model using each sampled set as training data, and select the optimal epoch using the same development data as before.
8. Use the trained AES model to evaluate the RMSE for the same test data as before.
9. Repeat Steps 2–8 five times and calculate the average RMSE for the test data.

**Fig. 13:**
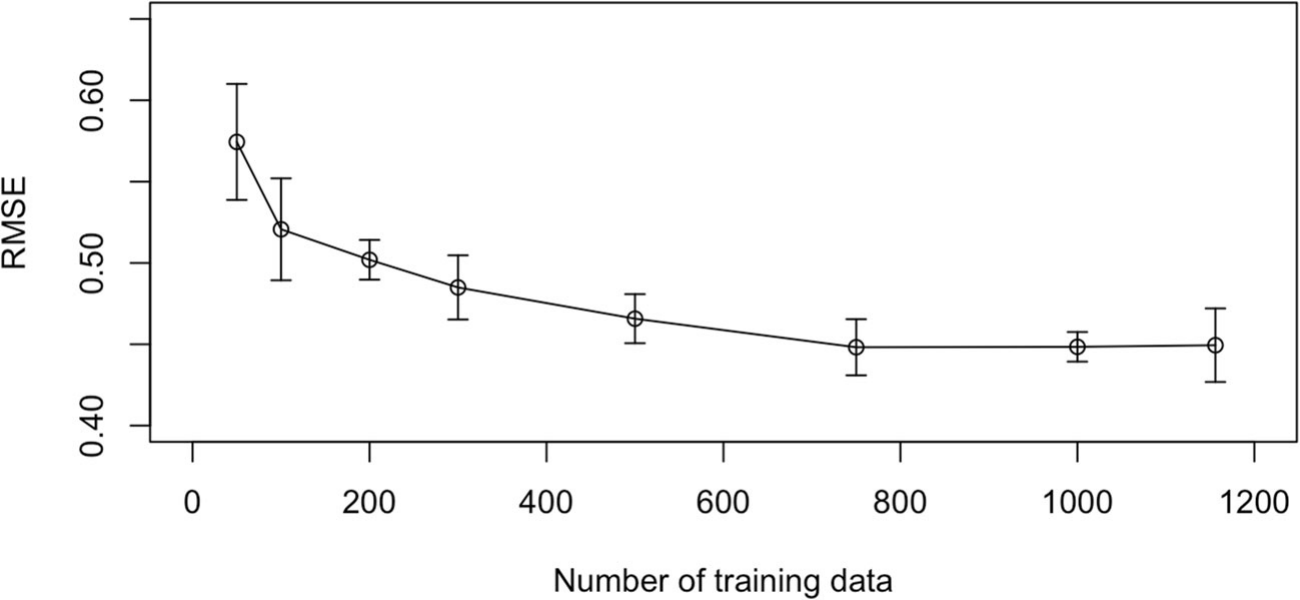
Relationship between the number of training samples and the ability prediction accuracy of AES

**Fig. 14:**
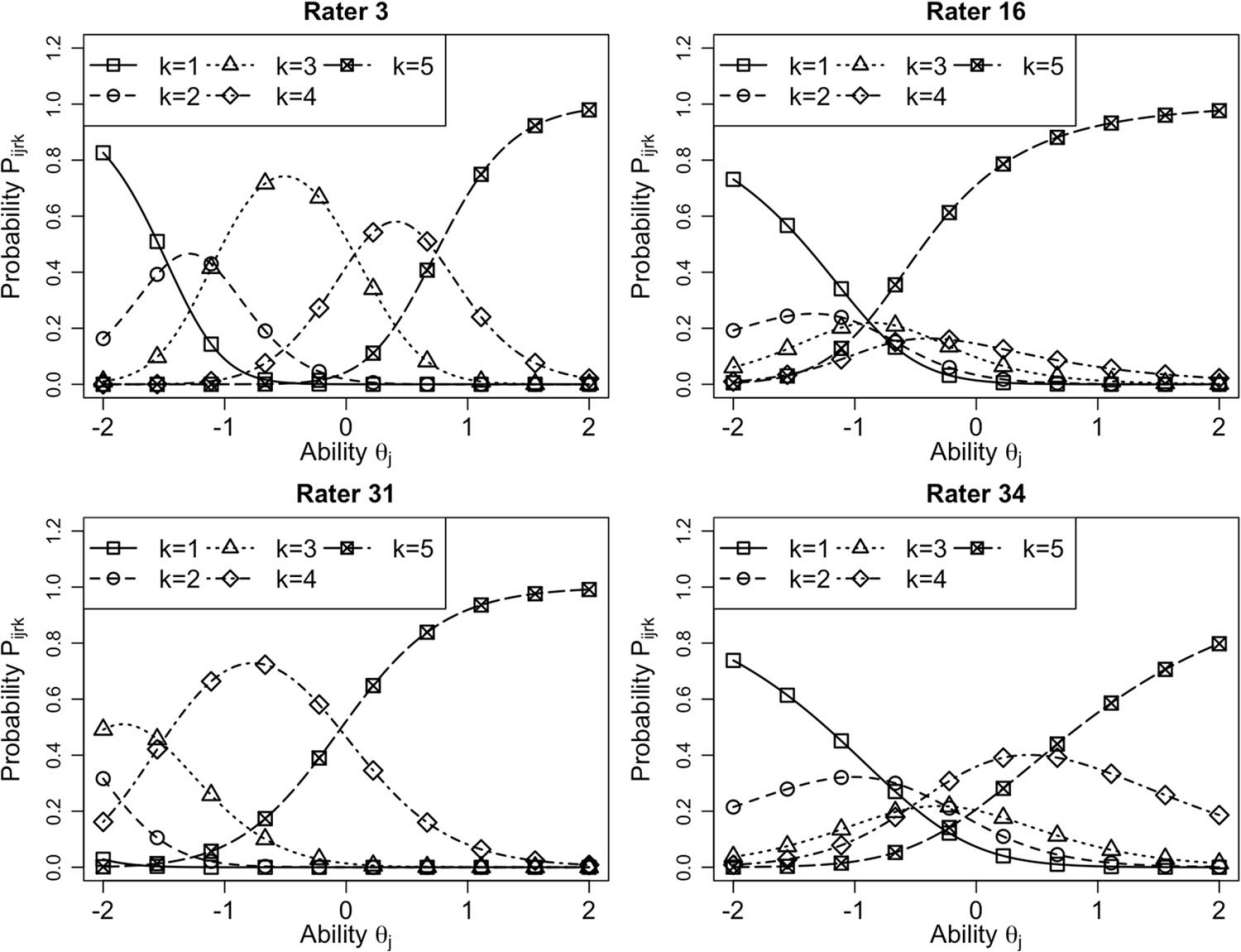
Item response curves of four representative raters found in experiments using actual data

Figure 13 displays the results. The horizontal axis represents the number of training samples, and the vertical axis shows the RMSE values. Each plot illustrates the average RMSE, with error bars indicating the SD ranges. The results demonstrate that larger sample sizes enhance the accuracy of the AES model. Furthermore, while the RMSE decreases significantly when the sample size is small, the improvements tend to plateau beyond 500 samples. This suggests that, for this dataset, approximately 500 samples would be sufficient to train the AES model with reasonable accuracy. However, note that the required number of samples may vary depending on the essay tasks. A detailed analysis of the relationship between the required number of samples and the characteristics of essay writing tasks is planned for future work.

An inherent cost associated with the proposed method is the computational expense required to construct the BERT-based AES model. Specifically, a computer with a reasonably powerful GPU is necessary to efficiently train the AES model. In this study, for example, we utilized an NVIDIA Tesla T4 GPU on Google Colaboratory. To elaborate on the computational expense, we calculated the computation times and costs for the above experiment under a condition where 1155 training samples were used. Consequently, training the AES model with 1155 samples, including evaluating the RMSE for the development set of 289 essays in each epoch, took approximately 10 min in total. Moreover, it required about 10 s to predict the abilities of 361 examinees from their essays using the trained model. The computational units consumed on Google Colaboratory for both training and inference amounted to 0.44, which corresponds to approximately $0.044. These costs and the time required are significantly smaller than what is required for human scoring.

### Analysis of rater characteristics

The *MCMC statistics and model fitting* section demonstrated that the GMFM provides a better fit to the actual data compared with the MFRM and MFRM with RSS. To explain this, Table 13 shows the rater parameters estimated by the GMFM using the entire dataset. Additionally, Fig. 14 illustrates the item response curves (IRCs) for raters 3, 16, 31, and 34, where the horizontal axis represents the ability $\theta _j$, and the vertical axis depicts the response probability for each category.

The table and figure reveal that the raters exhibit diverse and unique characteristics in terms of severity, consistency, and range restriction. For instance, Rater 3 demonstrates nearly average values for all parameters, indicating standard rating characteristics. In contrast, Rater 16 exhibits a pronounced extreme response tendency, as evidenced by higher $d_{r2}$ and lower $d_{r5}$ values. Additionally, Rater 31 is characterized by a low severity score, generally preferring higher scores (four and five). Rater 34 exhibits a low consistency value $\alpha _r$, which results in minimal variation in response probabilities among categories. This indicates that the rater is likely to assign different ratings to essays of similar quality.

As detailed in the *Item Response Theory* section, the GMFM can capture these variations in rater severity, consistency, and range restriction simultaneously, while the MFRM and MFRM with RSS can consider only its subsets. We can infer that this capability, along with the large variety of rater characteristics, contributed to the superior model fit of the GMFM compared with the other models.

It is important to note that, the proposed method is also useful for facilitating linking for MFRM and MFRM with RSS, even though the model fits for them were relatively worse, as well as for the GMFM, which we mentioned earlier and is shown in Appendix B.

### Effect of using cloud workers as raters

As we detailed in the *Actual data* section, we used scores given by untrained non-expert cloud workers instead of expert raters. A concern with using raters from cloud workers without adequate training is the potential for greater variability in rating characteristics compared with expert raters. This variability is evidenced by the diverse correlations between the raters’ scores and their ground truth, reported in the *Actual data* section, and the large variety of rater parameters discussed above. These observations suggest the importance of the following two strategies for ensuring reliable essay scoring when employing crowd workers as raters.

1. Assigning a larger number of raters to each essay than would typically be used with expert raters.
2. Estimating the standardized essay scores while accounting for differences in rater characteristics, potentially through the use of IRT models that incorporate rater parameters, which we used in this study.

## Conclusion

In this study, we propose a novel IRT-based linking method for essay-writing tests that uses AES technology to enable parameter linking based on IRT models with rater parameters across multiple groups in which neither examinees nor raters are shared. Specifically, we use a deep neural AES method capable of predicting IRT-based examinee abilities based on their essays. The core concept of our approach involves developing an AES model to predict examinee abilities using data from a reference group. This AES model is then applied to predict the abilities of examinees in the focal group. These predictions are used to estimate the linking coefficients required for linear linking. Experimental results with real data demonstrate that our method successfully accomplishes test linking with accuracy comparable to that of linear linking using few common examinees.

In our experiments, we compared the linking performance of the proposed method with linear linking based on the mean and sigma method using only five or ten common examinees. However, such a small number of common examinees is generally insufficient for accurate linear linking and thus leads to unstable estimation of linking coefficients, as discussed in the “Analysis of the linking method based on common examinees” section. Although this study concluded that our method could perform linking with accuracy comparable to that of linear linking using few common examinees, further detailed evaluations of our method involving comparisons with various conventional linking methods using different numbers of common examinees and raters will be the target of future work.

Additionally, our experimental results suggest that although the AES model may not provide sufficient predictive accuracy for individual examinee abilities, it does tend to yield reasonable mean and SD values for the ability distribution of focal groups. This lends credence to our assumption stated in the *Proposed method* section that AES models incorporating IRT can offer valuable insights into differences in ability distribution across various groups, thereby validating their utility for test linking. This result also supports the use of the mean and sigma method for linking. While concurrent calibration, another common linking method, requires highly accurate individual AES-predicted abilities to serve as anchor values, linear linking through the mean and sigma method necessitates only the mean and SD of the ability distribution. Given that the AES model can provide accurate estimates for these statistics, successful linking can be achieved, as shown in our experiments.

A limitation of this study is that our method is designed for test situations where a single essay writing item is administered to multiple groups, each comprising different examinees and raters. Consequently, the method is not directly applicable for linking multiple tests that offer different items. Developing an extension of our approach to accommodate such test situations is one direction for future research. Another involves evaluating the effectiveness of our method using other datasets. To the best of our knowledge, there are no open datasets that include examinee essays along with scores from multiple assigned raters. Therefore, we plan to develop additional datasets and to conduct further evaluations. Further investigation of the impact of the AES model’s accuracy on linking performance is also warranted.

## Data Availability

The data and materials from our experiments are available at https://github.com/AI-Behaviormetrics/LinkingIRTbyAES.git. This includes all experimental results and a sample dataset.

## Appendix A: Data splitting procedures

In this appendix, we explain the detailed procedures used to construct the reference group and the focal group while aiming to ensure distinct distributions of examinee abilities and rater severities, as outlined in experimental Procedure 2 in the *Experimental procedures* section.

**Table 14:** Detailed data separation conditions

		Ability		Severity	
	Groups	Mean $\acute{\mu }_{\theta }$	SD $\acute{\sigma }_{\theta }$	Mean $\acute{\mu }_{\beta }$	SD $\acute{\sigma }_{\beta }$
Condition 1	Reference	$\mu ^{\text {all}}_\theta + 0.5 \sigma ^{\text {all}}_\theta$	1.0	$\mu ^{\text {all}}_\beta - 0.5 \sigma ^{\text {all}}_\beta$	1.0
	Focal	$\mu ^{\text {all}}_\theta - 0.5 \sigma ^{\text {all}}_\theta$	1.0	$\mu ^{\text {all}}_\beta + 0.5 \sigma ^{\text {all}}_\beta$	1.0
Condition 2	Reference	$\mu ^{\text {all}}_\theta + 0.5 \sigma ^{\text {all}}_\theta$	1.0	$\mu ^{\text {all}}_\beta - 0.5 \sigma ^{\text {all}}_\beta$	1.0
	Focal	$\mu ^{\text {all}}_\theta - 0.5 \sigma ^{\text {all}}_\theta$	0.5	$\mu ^{\text {all}}_\beta + 0.5 \sigma ^{\text {all}}_\beta$	0.5
Condition 3	Reference	$\mu ^{\text {all}}_\theta + 0.5 \sigma ^{\text {all}}_\theta$	1.0	$\mu ^{\text {all}}_\beta + 0.5 \sigma ^{\text {all}}_\beta$	1.0
	Focal	$\mu ^{\text {all}}_\theta - 0.5 \sigma ^{\text {all}}_\theta$	1.0	$\mu ^{\text {all}}_\beta - 0.5 \sigma ^{\text {all}}_\beta$	1.0
Condition 4	Reference	$\mu ^{\text {all}}_\theta + 0.5 \sigma ^{\text {all}}_\theta$	1.0	$\mu ^{\text {all}}_\beta + 0.5 \sigma ^{\text {all}}_\beta$	1.0
	Focal	$\mu ^{\text {all}}_\theta - 0.5 \sigma ^{\text {all}}_\theta$	0.5	$\mu ^{\text {all}}_\beta - 0.5 \sigma ^{\text {all}}_\beta$	1.0
Condition 5	Reference	$\mu ^{\text {all}}_\theta$	1.0	$\mu ^{\text {all}}_\beta$	1.0
	Focal	$\mu ^{\text {all}}_\theta + \sigma ^{\text {all}}_\theta$	0.5	$\mu ^{\text {all}}_\beta + \sigma ^{\text {all}}_\beta$	0.5
Condition 6	Reference	$\mu ^{\text {all}}_\theta$	1.0	$\mu ^{\text {all}}_\beta$	1.0
	Focal	$\mu ^{\text {all}}_\theta + 0.5 \sigma ^{\text {all}}_\theta$	0.5	$\mu ^{\text {all}}_\beta + 0.5 \sigma ^{\text {all}}_\beta$	0.5
Condition 7	Reference	$\mu ^{\text {all}}_\theta$	1.0	$\mu ^{\text {all}}_\beta$	1.0
	Focal	$\mu ^{\text {all}}_\theta - 0.5 \sigma ^{\text {all}}_\theta$	0.5	$\mu ^{\text {all}}_\beta - 0.5 \sigma ^{\text {all}}_\beta$	0.5
Condition 8	Reference	$\mu ^{\text {all}}_\theta$	1.0	$\mu ^{\text {all}}_\beta$	1.0
	Focal	$\mu ^{\text {all}}_\theta + 0.5 \sigma ^{\text {all}}_\theta$	0.5	$\mu ^{\text {all}}_\beta - 0.5 \sigma ^{\text {all}}_\beta$	0.5

*Note:* SD stands for standard deviation

Let $\mu ^{\text {all}}_\theta$ and $\sigma ^{\text {all}}_\theta$ be the mean and SD of the examinees’ abilities estimated from the entire dataset in Procedure 1 of the *Experimental procedures* section. Similarly, let $\mu ^{\text {all}}_\beta$ and $\sigma ^{\text {all}}_\beta$ be the mean and SD of the rater severity parameter estimated from the entire dataset. Using these values, we set target mean and SD values of abilities and severities for both the reference and focal groups. Specifically, let $\acute{\mu }^{\text {ref}}_{\theta }$ and $\acute{\sigma }^{\text {ref}}_{\theta }$ denote the target mean and SD for the abilities of examinees in the reference group, and $\acute{\mu }^{\text {ref}}_{\beta }$ and $\acute{\sigma }^{\text {ref}}_{\beta }$ be those for the rater severities in the reference group. Similarly, let $\acute{\mu }^{\text {foc}}_{\theta }$, $\acute{\sigma }^{\text {foc}}_{\theta }$, $\acute{\mu }^{\text {foc}}_{\beta }$, and $\acute{\sigma }^{\text {foc}}_{\beta }$ represent the target mean and SD for the examinee abilities and rater severities in the focal group. Each of the eight conditions in Table 1 uses these target values, as summarized in Table 14.

Given these target means and SDs, we constructed the reference and focal groups for each condition through the following procedure.

1. Prepare the entire set of examinees and raters along with their ability and severity estimates. Specifically, let $\hat{\varvec{\theta }}$ and $\hat{\varvec{\beta }}$ be the collections of ability and severity estimates, respectively.
2. Randomly sample a value from the normal distribution $N(\acute{\mu }^{\text {ref}}_\theta , \acute{\sigma }^{\text {ref}}_\theta )$, and choose an examinee with $\hat{\theta }_j \in \hat{\varvec{\theta }}$ nearest to the sampled value. Add the examinee to the reference group, and remove it from the remaining pool of examinee candidates $\hat{\varvec{\theta }}$.
3. Similarly, randomly sample a value from $N(\acute{\mu }^{\text {ref}}_\beta ,\acute{\sigma }^{\text {ref}}_\beta )$, and choose a rater with $\hat{\beta }_j \in \hat{\varvec{\beta }}$ nearest to the sampled value. Then, add the rater to the reference group, and remove it from the remaining pool of rater candidates $\hat{\varvec{\beta }}$.
4. Repeat Steps 2 and 3 for the focal group, using $$N(\acute{\mu }^{\text {foc}}_\theta , $$$$\acute{\sigma }^{\text {foc}}_\theta )$$ and $$N(\acute{\mu }^{\text {foc}}_\beta ,\acute{\sigma }^{\text {foc}}_\beta )$$ as the sampling distributions.
5. Continue to repeat Steps 2, 3, and 4 until the pools $\hat{\varvec{\theta }}$ and $\hat{\varvec{\beta }}$ are empty.
6. Given the examinees and raters in each group, create the data for the reference group $\textbf{U}^{\text {ref}}$ and the focal group $\textbf{U}^{\text {foc}}$.
7. Remove examinees from each group, as well as their data, if they have received scores from only one rater, thereby ensuring that each examinee is graded by at least two raters.

## Appendix B: Experimental results for MFRM and MFRM with RSS

**Table 15:** RMSEs of the different experimental conditions for MFRM

		Conditions							
		1	2	3	4	5	6	7	8
Unlinked	$\theta _j$	0.85	0.82	0.87	0.83	0.69	0.49	0.43	0.46
	$\beta _r$	0.88	0.82	0.89	0.77	0.71	0.59	0.27	0.56
	$d_{r2}$	0.12	0.51	0.13	0.41	0.19	0.16	0.33	0.12
	$d_{r3}$	0.05	0.20	0.04	0.19	0.13	0.22	0.14	0.16
	$d_{r4}$	0.04	0.16	0.05	0.12	0.11	0.10	0.11	0.12
	$d_{r5}$	0.14	0.55	0.12	0.47	0.07	0.33	0.36	0.29
Linked by	$\theta _j$	0.50	0.42	0.46	0.36	0.29	0.28	0.33	0.28
proposed method	$\beta _r$	0.49	0.42	0.45	0.32	0.15	0.14	0.26	0.13
	$d_{r2}$	0.17	0.24	0.18	0.17	0.36	0.20	0.16	0.18
	$d_{r3}$	0.04	0.11	0.08	0.10	0.08	0.15	0.05	0.09
	$d_{r4}$	0.06	0.07	0.07	0.07	0.16	0.12	0.07	0.16
	$d_{r5}$	0.15	0.28	0.18	0.20	0.16	0.18	0.16	0.07
Linked by	$\theta _j$	0.64	0.38	0.55	0.55	0.41	0.46	0.41	0.50
five common examinees	$\beta _r$	0.56	0.27	0.49	0.55	0.34	0.33	0.32	0.45
	$d_{r2}$	0.60	0.42	0.45	0.53	0.33	0.31	0.27	0.34
	$d_{r3}$	0.20	0.15	0.12	0.16	0.14	0.22	0.09	0.24
	$d_{r4}$	0.21	0.15	0.16	0.18	0.12	0.13	0.11	0.14
	$d_{r5}$	0.60	0.41	0.42	0.52	0.29	0.44	0.26	0.46
Linked by	$\theta _j$	0.46	0.32	0.45	0.43	0.36	0.39	0.42	0.38
ten common examinees	$\beta _r$	0.36	0.20	0.37	0.37	0.26	0.25	0.40	0.27
	$d_{r2}$	0.44	0.36	0.39	0.38	0.22	0.18	0.43	0.22
	$d_{r3}$	0.16	0.15	0.09	0.16	0.16	0.21	0.16	0.17
	$d_{r4}$	0.13	0.12	0.13	0.13	0.10	0.10	0.15	0.12
	$d_{r5}$	0.48	0.39	0.34	0.43	0.22	0.28	0.44	0.31

**Table 16:** RMSEs of the different experimental conditions for MFRM with RSS

		Conditions							
		1	2	3	4	5	6	7	8
Unlinked	$\theta _j$	0.82	0.79	0.84	0.84	0.68	0.47	0.41	0.47
	$\beta _r$	0.86	0.79	0.87	0.82	0.66	0.52	0.24	0.50
	$d_{r2}$	0.17	0.39	0.15	0.36	0.42	0.32	0.35	0.39
	$d_{r3}$	0.15	0.26	0.14	0.24	0.29	0.34	0.31	0.34
	$d_{r4}$	0.15	0.20	0.14	0.18	0.25	0.24	0.22	0.26
	$d_{r5}$	0.22	0.47	0.19	0.43	0.16	0.31	0.35	0.27
Linked by	$\theta _j$	0.52	0.34	0.51	0.36	0.29	0.28	0.31	0.29
proposed method	$\beta _r$	0.54	0.32	0.55	0.35	0.16	0.17	0.21	0.16
	$d_{r2}$	0.24	0.23	0.31	0.18	0.54	0.37	0.26	0.44
	$d_{r3}$	0.15	0.18	0.17	0.15	0.23	0.27	0.24	0.27
	$d_{r4}$	0.13	0.14	0.16	0.12	0.27	0.24	0.18	0.26
	$d_{r5}$	0.28	0.29	0.32	0.21	0.25	0.18	0.21	0.19
Linked by	$\theta _j$	0.68	0.42	0.66	0.36	0.50	0.36	0.47	0.38
five common examinees	$\beta _r$	0.58	0.33	0.62	0.25	0.35	0.28	0.40	0.25
	$d_{r2}$	0.69	0.38	0.56	0.40	0.51	0.45	0.52	0.51
	$d_{r3}$	0.36	0.23	0.32	0.23	0.41	0.32	0.38	0.32
	$d_{r4}$	0.32	0.19	0.27	0.19	0.28	0.25	0.28	0.28
	$d_{r5}$	0.76	0.44	0.63	0.42	0.50	0.39	0.55	0.39
Linked by	$\theta _j$	0.38	0.38	0.37	0.37	0.35	0.29	0.38	0.33
ten common examinees	$\beta _r$	0.28	0.29	0.23	0.31	0.23	0.17	0.32	0.25
	$d_{r2}$	0.33	0.45	0.32	0.44	0.36	0.37	0.40	0.40
	$d_{r3}$	0.20	0.26	0.22	0.25	0.35	0.28	0.29	0.34
	$d_{r4}$	0.20	0.22	0.17	0.19	0.24	0.25	0.21	0.27
	$d_{r5}$	0.36	0.51	0.39	0.49	0.28	0.27	0.39	0.28

The experiments discussed in the main text focus on the results obtained from GMFM, as this model demonstrated the best fit to the dataset. However, it is important to note that our linking method is not restricted to GMFM and can also be applied to other models, including MFRM and MFRM with RSS. Experiments involving these models were carried out in the manner described in the *Experimental procedures* section, and the results are shown in Tables 15 and 16. These tables reveal trends similar to those observed for GMFM, validating the effectiveness of our linking method under the MFRM and MFRM with RSS as well.

## Footnotes

1 The original paper referred to this model as the generalized MFRM. However, in this paper, we refer to it as GMFM because it does not strictly belong to the family of Rasch models.

2 https://pytorch.org/

## Code availability

The source code for our linking method, developed in R and Python, is available in the same GitHub repository.

## Open Practices Statement

All results presented from our experiments for all models, including MFRM, MFRM with RSS, and GMFM, as well as the results for each repetition, are available for download at https://github.com/AI-Behaviormetrics/LinkingIRTbyAES.git. This repository also includes programs for performing our linking method, along with a sample dataset. These programs were developed using R and Python, along with RStan and PyTorch. Please refer to the README file for information on program usage and data format details.
